# Efficacy of nucleos(t)ide analogues(NAs) in preventing virus reactivation in oncology patients with HBV infection after chemotherapy or surgery: A network meta-analysis

**DOI:** 10.3389/fonc.2022.1050714

**Published:** 2023-01-16

**Authors:** Yuqing Zhao, Yingying Song, Huan Zhang, Tongshuo Qu, Malina Axinbai, Yidian Yang, Liping Zhang

**Affiliations:** ^1^ Graduate School, Beijing University of Chinese Medicine, Beijing, China; ^2^ Department of Gastroenterology, Dongfang Hospital, Beijing University of Chinese Medicine, Beijing, China

**Keywords:** HBV reactivation, cancer patients, antiviral therapy, survival rate, chemotherapy disruption, network meta-analysis

## Abstract

**Objective:**

In this study, we aimed to perform a network meta-analysis to compare the effectiveness of NAs in decreasing the reactivation of HBV, reducing chemotherapy disruption, and improving survival in oncology patients.

**Methods:**

Relevant randomized controlled trials (RCT) evaluating the impact of NAs in HBV infected-related oncology patients were retrieved from electronic databases. The outcome indicators included reactivation rate, survival rate of 1 to 3 years after treatment, and chemotherapy disruption rate. The studies were evaluated for bias using the RCT risk of bias assessment tool recommended in the Cochrane Handbook. The risk ratio (RR) was used to compare the outcome indicators for the anti-viral treatment, and the surface under the cumulative ranking curves (SUCRA) was used to identify the optimal therapeutic regime.

**Results:**

A total of 67 trials containing 5722 patients were included in this study. Regarding the reduction of reactivation rate, entecavir, lamivudine, adefovir alone were less effective than the combination of lamivudine and entecavir (94.9%), with RR values ranging from 3.16 to 3.73. However, based on SUCRA, the efficacy of telbivudine (80.3%) and the combination of lamivudine and adefovir dipivoxil (58.8%) were also acceptable. Entecavir (RR values ranging from 1.25 to 1.50) and lamivudine (RR values ranging from 1.27 to 1.35) can prolong the survival rate of patients at 1-3 years, and were better than adefovir dipivoxil in the comparison of 1-year survival rate. The RR values were 1.18 and 1.19, respectively. And entecavir ‘s ranking in SUCRA was more stable. Entecavir, lamivudine, and tenofovir all reduced chemotherapy interruption rates compared with no antiviral therapy, especially for tenofovir.

**Conclusions:**

Current evidence shows that lamivudine combined with entecavir, telbivudine, and lamivudine combined with adefovir dipivoxil were the most effective in preventing virus reactivation in HBV infected-related cancer patients treated with chemotherapy. Entecavir had the most stable effect on survival, while tenofovir had the best impact on reducing the chemotherapy disruption rate. Due to limited quality and quantity of the included studies, more high-quality studies are required to verify the above conclusions.

**Systematic review registration:**

PROSPEROI [https://www.crd.york.ac.uk/PROSPERO/index.php], identifier CRD4202250685.

## Introduction

Reactivation of the hepatitis B virus (HBV) is a common complication in cancer patients treated with chemotherapy or immunosuppressive therapy. It is estimated that about 4% to -68% of HBV infected-related patients are at risk of virus reactivation during the delivery of immunosuppressive therapy or chemotherapy for oncology patients (1), and the overall liver-related mortality rate from HBV reactivation reported in the literature was 5% (2). Earlier literature even documented that chemotherapy-induced HBV reactivation rates could be as high as 88% (3), with the resulting delayed initiation or premature termination of scheduled chemotherapy reducing cancer patient survival (4). Literature studies have also shown that the HBV replication rate is higher in patients treated with strong immunosuppressive therapies (5). As a result, cancer patients with HBV infection may benefit from prophylactic anti-viral treatment with nucleos(t)ide analogues (NAs) drugs (6). Preemptive antiviral therapy prior to chemotherapy was shown to reduce the risk of cancer chemotherapy discontinuation, virus reactivation rates, HBV-related hepatitis and HBV related disease mortality (5).

Several anti-viral drugs could be used to treat HBV infections, including lamivudine(LAM), entecavir(ETV) and tenofovir(TDF) (3, 6, 7). However, currently, there is no consensus on which drugs are the most effective at preventing viral reactivation as some infections may develop resistance to the drug (8). Currently, LAM and ETV are the most commonly used NAs (9, 10). The European Association for the Study of Liver Diseases guidelines recommended the use of TDF in 2017 (10). The Asia-Pacific Consensus on Chronic Hepatitis B (2012) suggested starting LAM one week prior to the delivery of immunosuppressive therapy or chemotherapy and continuing this treatment up to at least six months after the completion of chemotherapy (11). Conversely, the American Association for the Study of Liver Diseases does not recommend prophylactic NAs therapy since HBV-infected oncology patients treated with LAM tend to develop drug resistance (12). Although previous meta-analyses have shown that LAM prophylaxis in oncology patients receiving chemotherapy can significantly reduce the risk of HBV reactivation and HBV related mortality (3, 7). Moreover, current evidence for the efficacy of ETV and TDF is based on their application in chronic HBV infected patients.

Most of the current studies on the use of NAs therapies to prevent HBV reactivation are based on two-arm placebo studies, and there is a lack of head-to-head research. Although the traditional meta-analysis could be used for pairwise comparison of drugs, it cannot simultaneously compare various treatment measures. Therefore, in this study, we aimed to perform a network meta-analysis to integrate and analyze the results of individual studies to guide clinical practice on the use of NAs in HBV-infected oncology patents treated with chemotherapy and immunosuppressive therapy.

## Materials and methods

### Protocol and registration

This study was registered with PROSPERO, number CRD4202250685.Besides, the study was performed according to the Preferred Reporting Items for Systematic Reviews and Meta-Analyses (PRISMA) guidelines.

### Search strategy and literature inclusion criteria

Several electronic databases, including PubMed, Embase, Cochrane Library, Clinical-trials.gov, Web of Science, China National Knowledge Internet(https://chn.oversea.cnki.net/index/), WANFANG DATA(wanfangdata.com.cn), and VIP(www.cqvip.com) were searched to identify relevant randomized controlled trials (RCTs) published until December 9, 2022. No language restrictions were applied to the search. A combination of medical subject headings (MeSH) and keywords such as “hepatitis B”, “HBV”, “entecavir”, “lamivudine”, “Adefovir dipivoxil”, “Telbivudine”, “Tenofovir”, “ETV”, “LAM”, “ADV”, “LdT”, “TDF”, “reactivate” and “survival” were used to retrieve relevant articles from the electronic databases. **Supplementary Information 1** provide an example of the search strategy results retrieved from PubMed.

The studies were included in the network meta-analysis if they consisted of RCTs (irrespective of the blinding method) comparing the efficacy of chemotherapy with or without different antiviral drugs. Interventions with chemotherapy alone or chemotherapy combined with basic symptomatic treatment were defined as blank control groups. RCTs evaluating patients with HBV-related hepatocellular carcinoma(HCC), treated with surgery instead of chemotherapy, were also included in the analysis. Studies were excluded if the outcome indicators were not included or inconsistent and the research purpose and intervention measures were inconsistent with the study. Studies were also excluded if the sample size of either the experiment or control group was less than 10 cases and the research data were not collected rigorously. Any duplicate studies were also excluded.

### Outcome indicators

The primary outcome was the HBV reactivation rate. HBV reactivation was defined as a 10-fold or greater increase in HBV DNA level compared with baseline level, or an absolute increase of the HBV DNA level that exceeds 1×10^9^copies/ml or baseline HBV DNA negatives converted to positive (12–14). The second outcome was survival rate, which was defined as the overall survival rate of each group within the follow-up time of each study. The survival period was rounded up to the nearest year. If it was less than 1 year, such as 48 weeks, it would be recorded as 1 year, and if it was less than 2 years, such as 96 weeks, it would be recorded as 2 years. The third outcome was the chemotherapy disruption rate, defined as the premature termination of chemotherapy or the delay of at least seven days between chemotherapy cycles due to HBV reactivation or related hepatitis (15–17).

### Data screening and quality evaluation

Two researchers(YQ.Z and Y.S)conducted the literature screening and data extraction independently. During the preliminary screening, duplicated studies, editorials, abstracts, and literature that did not meet the study’s eligibility criteria were excluded. After the preliminary screening, the full-text articles were thoroughly reviewed. The two researchers(H.Z and T.Q) cross-checked the selected documents to be included in the meta-analysis. Any disagreement was resolved *via* a discussion with a third researcher(LP.Z). And the third researcher reviewed the selected articles to be included in the meta-analysis. Subsequently, the researchers(YQ.Z and H.Z)extracted the location of the study, types of cancer, age, sex, types of interventions, and outcome measures from the relevant studies. The researchers(YD.Y and M.A) evaluated the risk of bias in the included studies by using the Cochrane collaboration risk of bias tool (18, 19). This tool involves rating the level of bias as “high risk”, “low risk,” or ‘unclear’ based on the following criteria: randomization, treatment allocation concealment, blinding of participants, care providers and outcome assessors, drop-out rate, selective outcome reporting, similarity at baseline.

### Statistical analysis

The Stata version16.0 software was used to analyze and compare the studies. The relative risk ratio (RR) interval was used to estimate the count data, and the 95% confidence interval (95% CI) was used as the effect index. A 95% CI across 1 indicates no statistical difference. The global consistency and inconsistency of the data were tested by <*network meta i*> in Stata, and the local inconsistency was tested by node-splitting. For this analysis, a p-value below 0.05 was deemed statistically significant. The heterogeneity between studies was assessed using the *I-squared (I^2^) statistic*, whereby an *I^2^* test result above *50%* indicates significant heterogeneity between the studies. If significant heterogeneity was present among the included studies, additional subgroup analysis or meta-regression (<*metareg*>) or sensitivity analysis(<*metaninf*>) were performed to explore the source of the heterogeneity. The network structure was used to show the distribution and sample size of the direct comparison of the included original studies. The efficacy of each drug or combination of drugs for each outcome measure was ranked using the surface under the cumulative ranking curve (SUCRA). We used funnel plots and Egger tests to assess publication bias.

## Results

### Literature search and screening results

**Figure 1:**
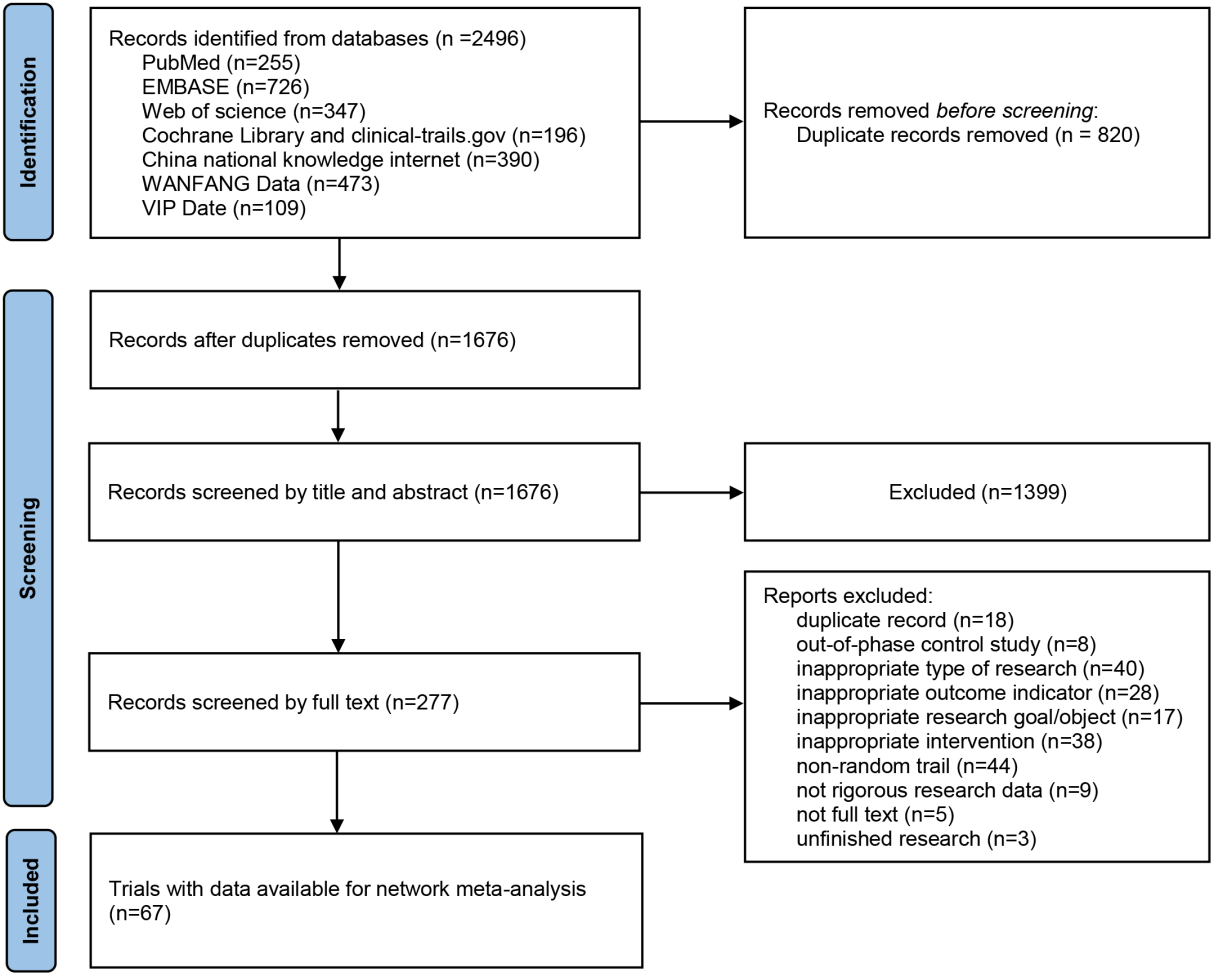
Study selection process.

A total of 2496 articles were initially screened based on the search terms, and 1676 articles remained after removing duplicates. 1399 articles were first excluded by reading the titles and abstracts. The remaining 277 articles were screened one by one by reading the full text, and 67 articles were finally included. The literature screening process is illustrated in **Figure 1**.

### Study characteristics and quality assessment

Of these 67 studies, four were from Korea, Turkey, Australia and Spain, and the remaining study population belonged to China. Among the included studies,41 studies (15, 17, 20–58) reported on the HBV reactivation rate, 25 studies (28, 29, 34, 43, 52, 59–78) reported the 1-year survival rate, 17 studies (28, 47, 60–62, 66–69, 71, 72, 74, 77–81) reported the 2-year survival rate, 10 studies (52, 62, 68–71, 74, 76, 80, 82) reported the 3-year survival rate, and 13 studies (15, 17, 21, 23, 25, 26, 32, 40, 46, 51, 55, 83, 84) reported the chemotherapy disruption rate. The cancers evaluated in the RCTs, included, 37 studies (15, 28, 29, 34, 38, 41, 43, 47, 48, 50, 52, 53, 59–82, 85) on hepatocellular carcinoma, 9 studies (26, 27, 35, 37, 40, 46, 49, 56, 84) on lymphoma, 3 studies (22, 24, 33) on breast cancer, 4 studies on hematological diseases (33, 42, 45, 57), 3 studies on nasopharyngeal carcinoma (21, 39, 55), 2 studies (17, 51) on lung cancer, and the remaining 9 studies (20, 23, 25, 32, 36, 44, 54, 58, 83) evaluated other cancers. A total of 5722 cases were included in this network meta-analysis, as shown in **Table S1**. The publication bias of the included studies is summarized in **Tables S2-S6**.

### HBV reactivation rate

**Figure 2:**
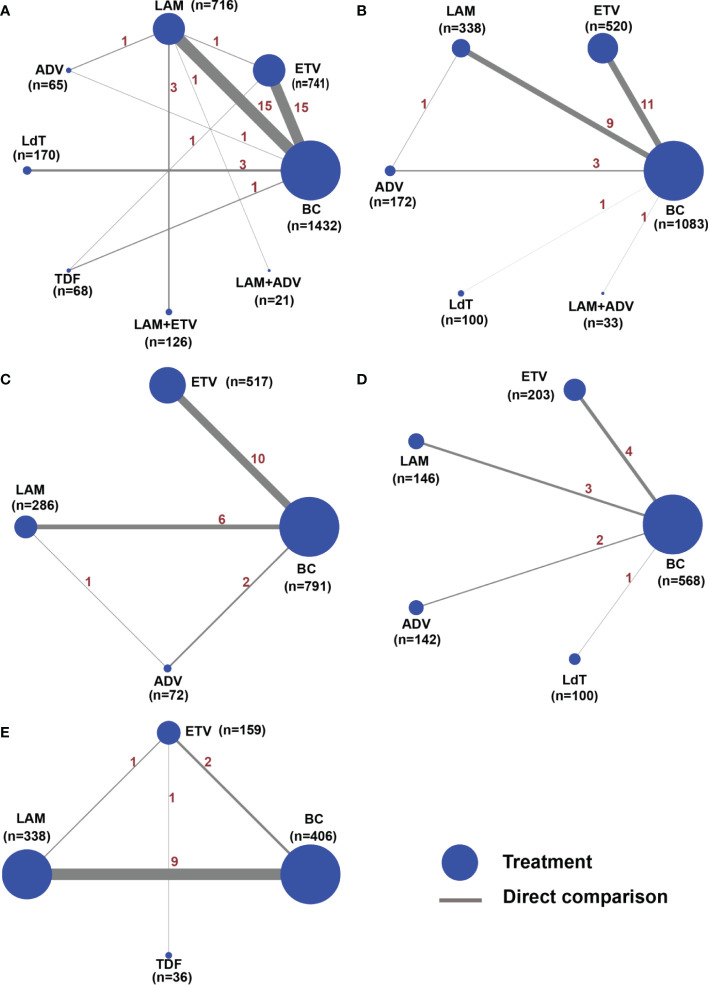
Network structure map for all outcome indicators. The network plots show a direct comparison of the different treatments, whereby the node size corresponds to the sample size. The thickness of solid lines reflects the number of studies included in the specific direct comparison for the following outcome indicators. **(A)** HBV reactivation rate, **(B)** 1-year survival rate, **(C)** 2-year survival rate, **(D)** 3-year survival rate, and **(E)** chemotherapy disruption rate.

Amongst the 41 studies evaluating the HBV reactivation rate, eight treatment measures were evaluated (**Figure 2A**), including the blank control group. All the seven types of anti-viral therapies evaluated were better than the blank control group in these studies. As we have shown, the *I^2^* value for the heterogeneity test was 18.5% (**Figure S1**) and the *P* value for the global inconsistency test was 0.3492 (**Table S7**). The local inconsistency among these studies was small (**Tables S8, 9** and **Figure S1**). In addition, subgroup analysis, meta-regression and sensitivity analysis demonstrated the stability of the results (**Figures S2-3** and **Table S10**).

**Figure 3:**
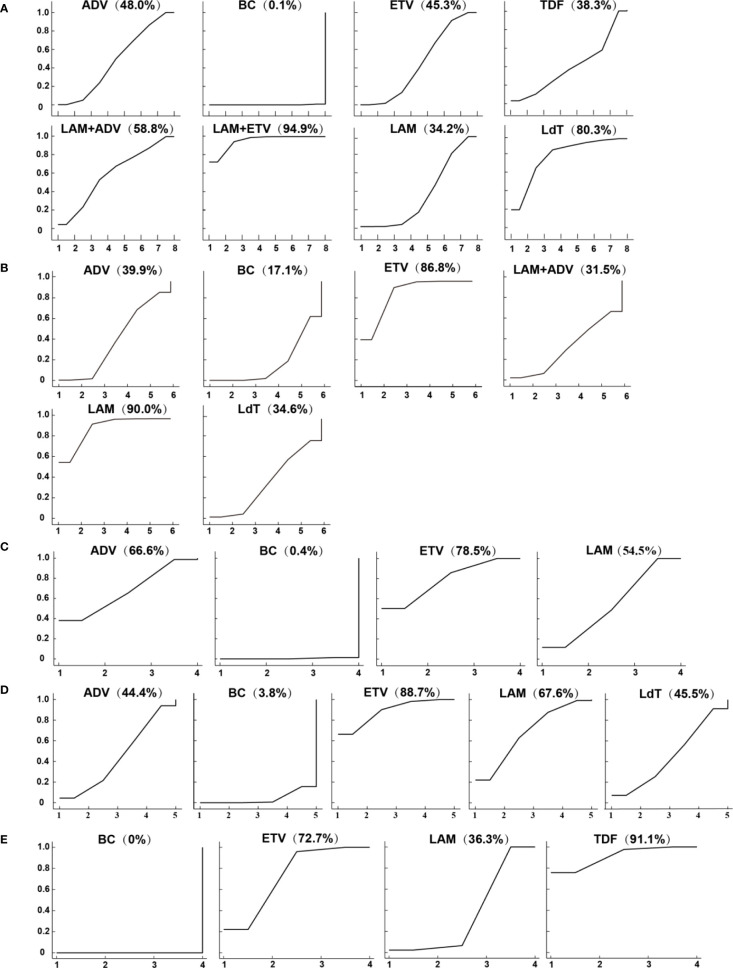
Surface under the cumulative ranking curves (SUCRAs) for **(A)** the HBV reactivation rate, **(B)** 1-year survival rate **(C)** 2-year survival rate **(D)** 3-year survival rate, and **(E)** chemotherapy disruption rate.

The results of the network meta-analysis showed that ETV (RR = 0.21, 95% CI (0.14 to 0.30)), LAM (RR = 0.23, 95% CI (0.17 to 0.32)), adefovir dipivoxil(ADV) (RR = 0.20, 95% CI (0.10 to 0.37)), telbivudine(LdT) (RR = 0.10, 95% CI (0.04 to 0.26)), TDF (RR = 0.24, 95% CI (0.07 to 0.78)), LAM combined with ETV (RR = 0.06, 95% CI (0.03 to 0.14)), and LAM combined with ADV (RR = 0.16, 95% CI (0.06 to 0.46)) reduced the HBV virus reactivation compared with perioperative or no antiviral prophylaxis before and after chemotherapy. Single agent ETV (RR = 3.31, 95% CI (1.36 to 8.04)), LAM (RR =3.73, 95% CI (1.78 to 7.83)) and ADV (RR = 3.16, 95% CI (1.23 to 8.13)) had a worse therapeutic effect when compared with LAM combined with ETV. The HBV reactivation rate did not vary significantly among the other anti-viral therapies (**Table 1A**). Based on the SUCRA ranking, LAM combined with ETV (94.9%), LdT (80.3%), and LAM combined with ADV(58.8%) had the best curative effect (**Figure 3A**).

**Table 1:** Pairwise comparison for each outcome indicator according to the network meta-analysis.

A																					
ETV																					
0.89 (0.54,1.45)	LAM																				
1.05 (0.50,2.18)	1.18 (0.66,2.13)					ADV															
2.07 (0.75,5.73)	2.34 (0.86,6.35)					1.98 (0.63,6.17)			LdT												
0.85 (0.27,2.66)	0.96 (0.28,3.27)					0.82 (0.21,3.11)			0.41 (0.09,1.87)			TDF									
3.31 (1.36,8.04)	3.73 (1.78,7.83)					3.16 (1.23,8.13)			1.60 (0.46,5.54)			3.87 (0.93,16.13)			LAM+ETV						
1.29 (0.42,3.90)	1.45 (0.54,3.93)					1.23 (0.39,3.90)			0.63 (0.15,2.55)			1.51 (0.31,7.27)			0.39 (0.11,1.34)				LAM+ADV		
0.21 (0.14,0.30)	0.23 (0.17,0.32)					0.20 (0.10,0.37)			0.10 (0.04,0.26)			0.24 (0.07,0.78)			0.06 (0.03,0.14)				0.16 (0.06,0.46)		BC
B																					
ETV																					
0.99 (0.86,1.13)		LAM																			
1.18 (1.01,1.36)		1.19 (1.02,1.39)					ADV														
1.20 (0.99,1.44)		1.21 (1.00,1.48)					1.02 (0.83,1.25)			LdT											
1.21 (0.97,1.51)		1.23 (0.98,1.54)					1.03 (0.81,1.30)			1.01 (0.78,1.31)			LAM+ADV								
1.25(1.14,1.37)		1.27 (1.14,1.41)					1.06 (0.94,1.21)			1.04 (0.89,1.23)			1.03 (0.85,1.26)							BC	
C																					
ETV																					
1.08 (0.88,1.33)				LAM																	
1.03 (0.75,1.42)				0.96 (0.71,1.28)							ADV										
1.44 (1.25,1.65)				1.33 (1.13,1.57)							1.39 (1.04,1.86)						BC				
D																					
ETV																					
1.12 (0.81,1.54)			LAM																		
1.25 (0.91,1.71)			1.12 (0.80,1.56)					ADV													
1.25 (0.87,1.78)			1.12 (0.78,1.61)					1.00 (0.70,1.43)						LdT							
1.50 (1.18,1.91)			1.35 (1.05,1.73)					1.21 (0.95,1.52)						1.21 (0.92,1.57)				BC			
E																					
ETV																					
0.53 (0.25,1.13)					LAM																
1.19 (0.74,1.92)					2.24 (0.91,5.51)						TDF										
0.17 (0.08,0.35)					0.31 (0.22,0.44)						0.14 (0.06,0.34)					BC					

The therapeutic effects are expressed as risk ratio (95% confidence interval) between interventions. **(A)** HBV reactivation rate (41 trials, 3339 participants), **(B)** 1-year survival rate (24 trials, 2246 participants), **(C)** 2-year survival rates (17 trials, 1666 participants), **(D)** 3-year survival rate (10 trials, 1159 participants), **(E)** chemotherapy disruption rate (13 trials, 939 participants).

### 1-year survival rate

Twenty-five studies reported the 1-year survival rate for ETV, LAM, ADV, LdT, and LAM combined with ADV versus the blank control group (**Figure 2B** and **Table 1B**). We used a random-effects model to test for heterogeneity between studies, and the results showed that *I^2^* was 53.7%, suggesting a significant heterogeneity(**Figure S4**). We then performed subgroup analysis and sensitivity analysis. First, subgroups were divided according to different control groups. The results showed that there was heterogeneity in the comparison of ETV and black control group(BC)(**Figure S5**). Combined with the sensitivity analysis, it suggested that the study ZX.F 2011 (61)、ZJ.W 2014 (65)、 W.T 2018 (76)、G.H 2018 (52) may bring mild heterogeneity(**Figure S6**). Then, after removing the above studies one by one, the heterogeneity test was carried out again, and it was found that the source of heterogeneity was ZX.F 2011 (61) (the *I^2^* were 46.5%、70.2%、53.1%、68.5% respectively). We subsequently excluded this study. The global inconsistency suggested that the p value was 0.5752, and the node-splitting also showed that there was no local inconsistency (**Tables S7-9**).

Among the five anti-viral regimens, only ETV and LAM could increase the survival rate within one year, which was better than that of the blank control group and slightly better than that of the ADV group. Compared with the blank control, the RR value and 95% CI were 1.25 (1.14 to 1.37) for ETV and 1.27 (1.14 to 1.41) for LAM. Compared with ADV, the RR value and 95% CI were 1.18 (1.01 to 1.36) for ETV and 1.19 (1.02 to 1.39) for LAM (**Table 1B**). LdT and LAM combined with ADV, which ranked among the top three in reducing the HBV reactivation rate, did not show a significant difference in this outcome indicator. The ranking of the efficacy of each is shown in **Figure 3B**. As expected, the 1-year survival rate for LAM (90.0%) and ETV (86.8%) were significantly higher than for other drugs, all of which were less than 40%.

### 2-year survival rate

Seventeen of the included studies reported the 2-year survival rates for ETV, LAM, and ADV (**Figure 2C** and **Table 1C**). All treatments were superior to the blank control group. Compared with the blank control, the RR value and 95% CI were 1.44 (1.25 to 1.65) for ETV, 1.33 (1.13 to 1.57) for LAM, and 1.39 (1.04 to 1.86) for ADV. The 2-year survival rate did not differ significantly between the three treatments. The ranking of the efficacy of each drug is illustrated in **Figure 3C**. ETV (78.5%) was still the most effective positive drug. There was no inconsistency among the studies, and heterogeneity was acceptable (**Tables S7-9** and **Figures S7-8**).

### 3-year survival rate

Ten of the included studies reported the 3-year survival rates for four anti-viral therapies: ETV, LAM, ADV, and LdT (**Figure 2D**). Only ETV (RR=1.50, 95%CI (1.18 to 1.91) and LAM (RR=1.35, 95%CI (1.05 to 1.73) resulted in a better 3-year survival when compared with the blank control group. There was no difference among the positive drugs. ADV and LdT still showed no superiority (**Table 1D**). The included studies were all two-arm comparisons with a blank control group, with no closed loop, and they directly fitted a consistency model. The heterogeneity between the studies was acceptable, as shown in **Tables S7-9** and **Figures S9-10**. The order of the efficacy of the four positive drugs is illustrated in **Figure 3D**. ETV (88.7%) and LAM (67.6%) remained stable, ranking first and second, respectively.

### Chemotherapy disruption rate

A total of 13 studies reported the chemotherapy disruption rate for three positive treatments, including ETV, LAM, and TDF (**Figure 2E**). All treatments were superior to the blank control group and resulted in an RR value and 95% CI of 0.17 (0.08 to 0.35) for ETV, 0.31 (0.22 to 0.44) for LAM, and 0.14 (0.06 to 0.34) for TDF (**Table 1E**). However, the chemotherapy disruption rate did not differ significantly between the three treatments. The treatment efficacy for each drug is illustrated in **Figure 3E**. TDF (91.1%) had the best efficacy, followed by ETV (72.7%) and LAM (36.3%). The studies showed no inconsistency and acceptable heterogeneity, as shown in **Tables S7-9** and **Figure S11, 12**.

### Assessment of publication bias

Funnel plots and Egger’s tests for all indicators showed the presence of publication bias. Therefore, we continued with the cut-and-patch method test using the command <*metatrim*>. The results for the reactivation rate showed no need for dummy filling of the study, and the results of the meta-analysis were unchanged, indicating that the results of the initial meta-analysis were stable and that publication bias did not affect the results. The results for the chemotherapy disruption rate, like the reactivation rate, did not require a dummy cut of the original study. Results for 1-3 year survival rates were subjected to a dummy study fill. For the 1-year survival rate, after 7 iterations with 13 dummy studies filled, the meta-analysis was rerun and the results showed a 95% CI of 0.019 to 0.146, which is still statistically different, indicating that the results of the original studies before filling were stable and not changed by the presence of publication bias. For 2-year survival, after 4 iterations, 8 were filled, and the results showed 95% confidence intervals of 0.138 to 0.267. 3-year survival, after 3 iterations, 4 were filled, and the 95% CI was 0.120 to 0.287. The statistical differences before and after filling the above 3-year survival dummy did not change, indicating that the results were all stable and publication bias did not affect the stability of the results (**Figures S13-14** and **Table S11 in Supplementary**).

## Discussion

Reactivation of the HBV is a common complication in oncology patients treated with chemotherapy or immunosuppressive therapy. However, there is still no consensus on the optimal anti-viral therapy for HBV infected individuals treated with chemotherapy or immunosuppressive therapy. Only randomized controlled trials were included in this manuscript and a network meta-analysis was performed, which evaluated the impact of various anti-viral therapies on the HBV reactivation rate, survival, and chemotherapy disruption rate in cancer patients treated with chemotherapy or immunosuppressive therapies. Since surgery may promote HBV replication in patients with HCC and lead to worse survival, we also included RCTs that evaluated HCC treated with surgery in this network meta-analysis as long as they assessed the same outcome indicators (30, 70, 85).

Our results showed that the efficacy of each intervention drug in reducing the HBV reactivation rate was better than the no antiviral treatment, and the combination of LAM and ETV had the best efficacy. The HBV reactivation rate of LdT did not differ significantly from the other single drugs. However, the SUCRA showed that LdT had the second-best efficacy. Moreover, it was not inferior to the combination of LAM and ETV in a pairwise comparison, and the SUCRA analysis showed the stable response. In terms of improving the survival rate of patients, LAM and ETV had the best survival. After a 1-year follow-up, ETV and LAM were better than ADV in improving the survival rate, while the ADV only showed an advantage over the blank control group in the 2-year follow-up study. LdT and LAM combined with ADV did not show advantages in this study. Due to the limited number of studies, the survival rates for the combination of LAM with ETV and TDF could not be evaluated. Entecavir has the highest 2-year and 3-year survival rates. After evaluating the impact of anti-viral therapies on reducing the chemotherapy disruption rates, TDF had the best effect, followed by ETV and LAM. However, TDF did not show an advantage in all other outcome indicators. The efficacy of LAM was less than satisfactory, with an area under the SUCRA curve of 36.3%. Due to the limited number of studies, the rest of the anti-viral treatments could not be compared, and therefore their effect on the chemotherapy disruption rate remains unknown.

A meta-analysis reported by MY. Zhang et al. (86) synthesized 52 RCTs and cohort studies with findings that were complementary to ours. Our study focused on a broader range of RCTs in order to further complement the therapeutic findings related to antiviral drugs.The reduction in hepatitis and HBV-related deaths with ETV reported in the study by MY. Zhang et al. (86) may also account for the highest probability of prolonged survival found with ETV in our study. The probability of reducing all-cause mortality (47%) found for LdT in MY. Zhang ‘s study (86) was close to ours, and the highest probability of 3-year survival for LdT in our study was 45.5%. Also, LdT was the single agent with the highest probability of reducing reactivation rate in our study. In addition, the study by Zhang et al. (86) indicated that TDF was the most effective in reducing reactivation, while our study found that it was the most effective in reducing chemotherapy interruption and delay. Therefore, all these complementary findings increase the confidence of physicians to apply different antiviral drugs appropriately in the future.

LAM works by inhibiting the synthesis and prolongation of the HBV-DNA chain, reducing the viral load, and reducing the hepatic inflammatory response, which in turn reduces fibrosis in the liver (87). ETV achieves an anti-viral effect by hindering the initiation, transcription, and synthesis of HBV-DNA replication, inhibiting the HBV-DNA reverse transcriptase and polymerase’s activities, and disrupting the synthesis, extension, and assembly of the positive HBV-DNA strand (57). This may be the reason why they can enhance liver function, reduce the level of HBV-DNA, prolong the survival rate of patients, and reduce the reactivation of HBV when used together. ADV has a low drug resistance rate and no cross-resistance with other NAs, and is comparable to LAM in efficacy (88). The combination of LAM and ADV had greater viral suppression and a lower risk of genotypic resistance (89, 90). Of course, this conclusion remains to be determined (88). In this manuscript, LAM combined with ADV, although robustly the third based on SUCRA, the efficacy of this combination did not show a statistical difference compared with other interventions and needs more high-quality studies to verify. The advantage of LdT is its potent antiviral efficacy and high seroconversion rate (88). This may be the reason why LdT is the most effective single drug. A retrospective cohort study explored the effects of TDF and ETV on the survival and recurrence rate of patients with HBV-related HCC. It was found that compared with ETV treatment, the recurrence rate of HCC was significantly lower, and the overall survival rate was higher in patients treated with TDF (91). Due to the lack of prospective literature on the reduction of the survival rate of patients with TDF in this study, its effects could not be compared, but we speculate that they should not be inferior to ETV. In clinical practice guidelines, ETV and TDF are similarly recommended as first-line NAs for chronic HBV due to their similar high anti-viral efficacy and low drug resistance (10, 12, 92). Our findings indicate that although the combination of drugs can significantly reduce the HBV reactivation rate, the impact on survival was limited. At the same time, it is important to acknowledge that relatively few studies evaluated the effect of anti-viral treatments on survival and chemotherapy disruption. Therefore, the current evidence only showed that when each positive drug was used alone, LdT was better at reducing HBV reactivation rate. ETV and LAM can prolong the survival rate of patients at 1-3 years, and was better than ADV in the comparison of 1-year survival rate. Combined with SUCRA, the efficacy of ETV was more stable. ETV, LAM, and TDF all reduced chemotherapy interruption rates compared with no antiviral therapy. And TDF was more efficient than ETV and LAM.

The strength of our current study was that we re-examined the available RCTs as extensively and comprehensively as possible. This study compared reactivation rates, year-by-year survival rates, and chemotherapy disruption rate to analyze the efficacy of different antiviral agents across multiple indicators. In this study, we comprehensively analyzed the heterogeneity and inconsistency among studies and conducted a thorough assessment of publication bias to reduce confounding and suspicion of conclusions.

The study has some limitations that have to be acknowledged. The current study mainly focused on LAM and ETV since there are currently limited studies on other drugs. The uneven sample size distribution may have influenced the outcomes of this meta-analysis. Since only a few studies reported on chemotherapy disruption rate and survival rate, it was not possible to make a comprehensive comparison of all positive drugs, which eventually limited the findings for this outcome measure. Unfortunately, the quality of the original literature was poor. Finally, the difference in the curative effect between every single drug was small, and the results were unstable.

As a result, the ranking of the drugs may change with a change in the sample size. In summary, our findings indicate that all anti-viral HBV therapies evaluated in this study reduced the HBV reactivation rate when compared with no treatment. Based on the current research evidence, the optimal combination to reduce the reactivation rate was the combination of LAM and ETV, while LdT was identified as the most effective single agent. ETV had the most stable effect on survival, while TDF had the best impact on reducing the chemotherapy disruption rate. These findings suggest that the optimal anti-viral therapy for oncology patients that are HBV infected should be tailor-made for the patient depending on the risk of having HBV reactivation, the expected survival, and the need to improve the effects of chemotherapy. However, it is important to note that the current results are inconclusive due to the mixed quality of the included studies and the low number of studies on survival and chemotherapy disruption rates. Therefore more RCTs are required to identify the optimal anti-viral therapy.

## Data availability statement

The original contributions presented in the study are included in the article/**Supplementary Material**. Further inquiries can be directed to the corresponding author.

## Author contributions

All authors contributed to the study conception and design. Acquisition, analysis and interpretation of data were performed by YZ, HZ, MA, TQ, and YY. Drafting of the manuscript were performed by YZ, YS, HZ, and YS. Critical revision of the manuscript for important intellectual content performed by YZ, YS, MA, and LZ. All authors read and approved the final manuscript.
