# Supplementary material for: Efficacy of nucleos(t)ide analogues(NAs) in preventing virus reactivation in oncology patients with HBV infection after chemotherapy or surgery: A network meta-analysis
Source: Front Oncol. 2023 Jan 16;12:1050714. doi: 10.3389/fonc.2022.1050714 (PMC9885183; doi:10.3389/fonc.2022.1050714)

**Efficacy of nucleos(t)ide analogues(NAs) in preventing virus reactivation in oncology patients with HBV infection after chemotherapy or surgery: a network meta-analysis**

**SUPPLEMENTAL INFORMATIONS**

# Supplementary Information 1. Example PubMed search strategy

| #1 Hepatitis B [MeSH Terms]  #2 hepatitis B [Title/Abstract] OR hep B [Title/Abstract] OR HBV [Title/Abstract]  #3 #1 OR #2  #4 "reactivat*"[Title/Abstract] OR "reinfect*"[Title/Abstract] OR "resurrect*"[Title/Abstract] OR "resurgence"[Title/Abstract] OR "recurrence"[Title/Abstract] OR "revival"[Title/Abstract] OR "revivification"[Title/Abstract] OR "Survival"[Title/Abstract] OR "Delayed chemotherapy"[Title/Abstract]  #5 Lamivudine [MeSH Terms] OR Telbivudine [MeSH Terms] OR Tenofovir [MeSH Terms] OR Emtricitabine [MeSH Terms]  #6 Entecavir [Title/Abstract] OR epivir* [Title/Abstract] OR BCH189 [Title/Abstract] OR GR109714X [Title/ Abstract] OR lamivudin* [Title/Abstract] OR zeffix [Title/Abstract] OR heptovir [Title/Abstract] OR heptodin [Title/Abstract] OR telbivudin* [Title/ Abstract] OR Tyzeka [Title/Abstract] OR Disoproxil Fumarate [Title/Abstract] OR Viread [Title/Abstract] OR 9-PMPA [Title/Abstract]  #7 adefovir depivoxil [Title/Abstract] OR Hepsera [Title/Abstract] OR Preveon [Title/Abstract] OR gs 840 [Title/Abstract] OR emtriva* [Title/Abstract] OR Coviracil [Title/Abstract]  #8 ETV [Title/Abstract] OR LAM [Title/Abstract] OR TDF [Title/Abstract] OR TAF [Title/Abstract] OR LdT [Title/Abstract] OR FTC [Title/Abstract] OR ADV [Title/Abstract]  #9 #5 OR #6 OR #7 OR #8  #10 "control*"[Title/Abstract] OR "contrast"[Title/Abstract]  #11 #3 AND #4 AND #9 AND #10 |
| --- |

# Supplementary Information 2. Details of the included study

Table S1 Summary of included studies and patient characteristics. Description of each included trial including population, setting, comparison treatments and outcome indicators.

| Study | Types of cancer | Country | N  (T/C) | Sex(M/F) | Mean(SD/SE) OR the range of age(T/C） | | Intervention measures（T/C） | | Outcome indicator | Drop-out（T/C） | Treatment dosage | Duration of follow up for the reactivation outcome |
| --- | --- | --- | --- | --- | --- | --- | --- | --- | --- | --- | --- | --- |
| Jang,2006 | hepatocellular carcinoma | Korea | 36/37 | 61/12 | 52.5(8.4) | 53.2(9.0) | LAM | BC | ①⑤ | 2/1 | 100mg/d | after chemotherapy |
| Zeng,2007 | other cancer | China | 19/19 | 21/17 | 22-60 | | LAM | BC | ⑤ | N/A | 100mg/d |  |
| Duan,2009 | lung cancer | China | 30/30 | 36/24 | 35-71 | | LAM | BC | ①⑤ | N/A | 100mg/d | after chemotherapy |
| Xu,2009 | hepatocellular carcinoma | China | 20/20 | 32/8 | 51.2(9.3) | 48.4(9.7) | LAM | BC | ② | N/A | 100mg/d |  |
| Zhuang,2009 | other cancer | China | 30/34 | 51/13 | 56.3 | 52.9 | LdT | BC | ① | N/A | 600mg/d | after chemotherapy |
| Xiong,2010 | nasopharyngeal carcinoma | China | 26/25 | 35/16 | 30-65 | | LAM | BC | ①⑤ | N/A | 100mg/d | after chemotherapy |
| Xu,2010 | breast Cancer | China | 30/27 | * | 47.2 | | LAM | BC | ① | Unclear description | 100mg/d | after chemotherapy |
| Zhan,2010 | hepatocellular carcinoma | China | 36/24 | 42/18 | 43.5 | | LAM | BC | ②③ | N/A | 100mg/d |  |
| Zhang,2010 | hepatocellular carcinoma | China | 31/27 | 46/12 | 40(13) | | LAM | BC | ④ | N/A | 100mg/d |  |
| Fang,2011 | hepatocellular carcinoma | China | 100/100 | 119/81 | 53.9(12.2) | | ETV | BC | ②③ | N/A | 0.5mg/d |  |
| Jin,2011 | other cancer | China | 50/50 | * | * | * | LAM | BC | ①⑤ | N/A | 100mg/d | after chemotherapy |
| Long,2011 | breast cancer | China | 21/21 | 0/42 | 20-62 | 29-64 | LAM | BC | ① | N/A | 100mg/d | after chemotherapy |
| Fang,2012 | hepatocellular carcinoma | China | 26/30 | 43/13 | 48.9(7.4) | 50.0(9.6) | LAM | BC | ②③④ | N/A | 100mg/d |  |
| Wu,2012 | other cancer | China | 50/50 | 62/38 | 60.8(27.3) | 58.5(23.6) | LAM | BC | ①⑤ | N/A | 100mg/d | after chemotherapy |
| Dai,2013 | hepatocellular carcinoma | China | 30/30 | 75/15 | 46(9.7) | 45(11.5) | LAM | BC | ①②③ | N/A | 100mg/d | 1 month after treatment |
| Dai,2013 | hepatocellular carcinoma | China | 30/30 | 75/15 | 44(10.5) | 45(11.5) | ADV | BC | ①②③ | N/A | 10mg/d | 1 month after treatment |
| Shao,2013 | hepatocellular carcinoma | China | 30/32 | * | * | * | LAM | BC | ①② | N/A | 100mg/d | 6 month after treatment |
| Zhang,2013 | hepatocellular carcinoma | China | 32/30 | 48/14 | 52.5(1.6) | | LAM | BC | ② | N/A | 100mg/d |  |
| Li,2014 | hematologic diseases | China | 30/30 | 33/27 | 49.0(0.3) | 51.0(0.5) | ETV | BC | ① | N/A | 0.5mg/d | after chemotherapy |
| Li,2014 | hepatocellular carcinoma | China | 35/30 | 58/7 | 54.3(22.3) | | LAM | BC | ②③ | N/A | 100mg/d |  |
| Wan,2014 | hepatocellular carcinoma | China | 33/33 | 45/21 | 52.0(6.9) | 53.0(6.8) | LAM+  ADV | BC | ② | N/A | 100mg/d, 10mg/d |  |
| Wang,2014 | hepatocellular carcinoma | China | 53/36 | 73/16 | 50.3（9.7） | 49.9（10.0） | ETV | BC | ②③ | N/A | 0.5mg/d |  |
| Xu,2014 | hepatocellular carcinoma | China | 43/42 | * | * | * | ETV | BC | ②③ | N/A | 0.5mg/d |  |
| Xu,2014 | hepatocellular carcinoma | China | 92/89 | 164/17 | 56.0  （11.7） | 55.1（11.8） | LAM | BC | ②③④ | N/A | 100mg/d |  |
| Hong,2015 | breast Cancer | China | 21/27 | 0/188 | 33-61 | 33-61 | LAM | BC | ① | N/A | 100mg/d | after chemotherapy |
| Hou,2015 | hepatocellular carcinoma | China | 65/65 | 75/55 | 57.8  （11.2） | 58.6（11.4） | LAM | BC | ③ | N/A | 100mg/d |  |
| Wang,2015 | hepatocellular carcinoma | China | 32/32 | 49/15 | 58.6（4.4） | 58.2（6.1） | ETV | BC | ①② | N/A | 0.5mg/d | 12 month after treatment |
| Cai,2015 | lymphoma | China | 21/47 | * | * | * | LAM+  ADV | LAM | ① | N/A | 100mg/d, 10mg/d | unclear |
| Ho,2015 | other cancer | China | 35/35 | 43/47 | 21-82 | 29-76 | LAM | ADV | ① | N/A | 100mg/d, 10mg/d | after chemotherapy |
| Yang,2016 | hepatocellular carcinoma | China | 40/40 | 52/28 | 47.5（5.9） | 47.6（4.4） | LAM | BC | ②③ | N/A | 100mg/d |  |
| Bai,2016 | hepatocellular carcinoma | China | 42/42 | 46/38 | 51.3（6.9） | 50.2（6.2） | ETV | BC | ②③④ | N/A | 0.5mg/d |  |
| Ren,2016 | nasopharyngeal carcinoma | China | 43/41 | * | * | * | LAM | BC | ① | N/A | 100mg/d | after chemotherapy |
| Yin,2016 | hepatocellular carcinoma | China | 28/32 | 41/19 | 53.2（11.6） | 52.1（10.4） | ETV | BC | ② | N/A | 0.5mgd |  |
| Chen,2017 | hematologic diseases | China | 38/38 | 44/32 | 18-68 | 19-70 | ETV | BC | ① | N/A | 0.5mg/d | after chemotherapy |
| Hu,2017 | hepatocellular carcinoma | China | 65/65 | 106/24 | 47.0（5.3） | 46.7（5.2） | ETV | BC | ②③④ | N/A | 0.5mg/d |  |
| Jiang,2017 | hepatocellular carcinoma | China | 38/38 | 63/13 | 49.8（5.5） | 50.7（5.5） | ETV | BC | ② | N/A | 0.5mg/d |  |
| Li,2017 | hepatocellular carcinoma | China | 56/56 | 68/44 | 60.3（1.2） | 60.0（1.2） | ETV | BC | ①② | N/A | 0.5mg/d | 12 month after treatment |
| Luo,2017 | other cancer | China | 41/41 | 44/38 | 44.8(1.8) | 43.1(2.3) | LAM+ETV | LAM | ① | N/A | 100mg/d,  0.5mg/d | after chemotherapy |
| Buti,2017 | hematologic diseases | Spain | 33/28 | 34/23 | 69.9(3.3) | | TDF | BC | ① | N/A | 300mg/d | Within 18 months of treatment |
| Liang,2018 | hepatocellular carcinoma | China | 33/33 | 37/29 | 48.3(2.5) | 48.6(2.4) | ETV | BC | ③④ | N/A | 0.5mg/d |  |
| Tang,2018 | hepatocellular carcinoma | China | 63/67 | 100/30 | 49.4(11.3) | 50.7(11.3) | ETV | BC | ②④ | N/A | 0.5mg/d |  |
| Zhan,2018 | hepatocellular carcinoma | China | 23/22 | 36/9 | 47.8(8.6) | 45.1(8.4) | ETV | BC | ① | N/A | 0.5mg/d | after chemotherapy |
| Zhang,2018 | lymphoma | China | 56/56 | 78/34 | 53.1(14.5) | 52.2(14.6) | ETV | BC | ① | N/A | 0.5mg/d | after chemotherapy |
| Zhang,2018 | hepatocellular carcinoma | China | 41/41 | 57/25 | 48.1(6.8) | | ETV | BC | ① | N/A | 0.5mg/d | end of follow-up |
| Zheng,2018 | lung cancer | China | 35/35 | 39/31 | 56.3(11.5) | 55.6(11.3) | ETV | BC | ①⑤ | N/A | 0.5mg/d | after chemotherapy |
| Huang,2018 | hepatocellular carcinoma | China | 100/100 | 173/27 | 52（-） | 51（-） | LdT | BC | ①②④ | N/A | 600mg/d | Within 60 months of treatment |
| Chen,2019 | other cancer | China | 35/35 | 40/30 | 42.8(1.7) | 43.7(1.9) | LAM+ETV | LAM | ① | N/A | 100mg/d,0.5mg/d | unclear |
| Li,2019 | nasopharyngeal carcinoma | China | 62/57 | * | * | * | ETV | BC | ①⑤ | N/A | 0.5mg/d | after chemotherapy |
| Qiu,2019 | hematologic diseases | China | 50/50 | 58/42 | 49.9(5.3 ) | 49.8(5.3) | LAM+ETV | LAM | ① | N/A | 100mg/d,0.5mg/d | 1 month after treatment |
| Yang,2019 | hepatocellular carcinoma | China | 40/40 | 44/36 | 64.2(7.6) | 63.0(8.2) | ETV | BC | ③ | 2/3 | 0.5mg/d |  |
| Liu,2019 | lymphoma | China | 95/95 | 108/82 | 28-86 | 23-85 | ETV | BC | ① | N/A | 0.5mg/d | after chemotherapy |
| Lu,2019 | hepatocellular carcinoma | China | 40/40 | 63/17 | 64.1(2.1) | 64.0(2.0) | ETV | BC | ①③ | N/A | 0.5mg/d |  |
| Toka,2020 | other cancer | Turkey | 37/35 | 64/56 | 23-85 | 35-83 | ETV | TDF | ① | N/A | 0.5mg/d,245mg/d | 1 year after treatment |
| Gao,2020 | hepatocellular carcinoma | China | 60/60 | 69/51 | 46.1(4.1) | 45.1(4.2) | ETV | BC | ②③ | N/A | 0.5mg/d |  |
| Fan,2017 | lymphoma | China | 26/24 | 30/20 | 45.0(8.0) | 46.0(7.0) | ETV | LAM | ①⑤ | N/A | 100mg/d,  0.5mg/d | after chemotherapy |
| Ren,2012 | lymphoma | China | 42/42 | 43/41 | 20-60 | 21-59 | LAM | BC | ①⑤ | N/A | 100mg/d | after chemotherapy |
| Wang,2014 | other cancer | China | 25/25 | 31/19 | 32-76 | 33-72 | LAM | BC | ①⑤ | N/A | 100mg/d | after chemotherapy |
| Wu,2015 | lymphoma | China | 32/32 | 36/28 | 47.5(0.6) | 47.8(0.6) | ETV | BC | ① | N/A | 0.5mg/d | after chemotherapy |
| Yang,2016 | lymphoma | China | 36/36 | 56/52 | 30-75 | 40-80 | LAM | BC | ①⑤ | N/A | 100mg/d | after chemotherapy |
| Zhang,2020 | lymphoma | China | 36/36 | 46/26 | 48.2(5.9) | 47.9(4.9) | TDF | ETV | ⑤ | N/A | 300mg/d,  0.5mg/d |  |
| Pan,2015 | hepatocellular carcinoma | China | 42/22 | 66/34 | 44.7(5.7) | 43.7(5.1) | ADV | BC | ②③④ | N/A | 10mg/d |  |
| Huang,2015 | hepatocellular carcinoma | Australia | 100/100 | 179/21 | 50.6(7.8) | 50.5(8.5) | ADV | BC | ②④ | 3/3 | 10mg/d |  |
| Huang,2013 | hepatocellular carcinoma | China | 40/44 | 69/15 | 50.6(7.9) | 50.0(9.3) | LdT | BC | ① | N/A | 600mg/d | 1 month after treatment |
| Gong,2016 | hepatocellular carcinoma | China | 66/108 | 150/24 | 49.9(9.7) | 49.7(12.2) | ETV | BC | ① | N/A | 0.5mg/d | after chemotherapy |
| Wang,2015 | hepatocellular carcinoma | China | 20/54 | 64/10 | 50(10) | 49(12) | ETV | BC | ① | N/A | 0.5mg/d | after chemotherapy |
| Li,2012 | lymphoma | China | 15/15 | 14/16 | 23-56 | 19-54 | LAM | BC | ① | N/A | 100mg/d | after chemotherapy |
| Wang,2018 | hepatocellular carcinoma | China | 51/47 | 82/16 | 57.4(8.3) | 58.7(8.3) | ETV | BC | ① | N/A | 0.5mg/d | 1 month after treatment |
| Hu,2017 | hepatocellular carcinoma | China | 43/43 | 45/41 | 42.8(3.5) | 42.3(3.7) | ETV | BC | ① | N/A | 0.5mg/d | Within 2 years of treatment |

①:reactivation rate; ②: 1 year-survival rate; ③: 1 year-survival rate; ④: 1 year-survival rate; ⑤:Chemotherapy disruption; T refers to the treatment group, C refers to the control group.

# Supplementary Information 3. Detailed risk of bias assessments for each included trial

Table S2 Reactivation rate:

| First Author, Year | Randomization | Blinding | Allocation concealment | Incomplete outcome data | Intent to treat analysis | Other sources of bias |
| --- | --- | --- | --- | --- | --- | --- |
| Jang ,2006 | Low Risk | Unclear | Unclear | Low Risk | Low Risk | Unclear |
| Duan,2009 | Unclear | Unclear | Unclear | Low Risk | Low Risk | Unclear |
| Zhuang,2009 | Unclear | Unclear | Unclear | Low Risk | Low Risk | Unclear |
| Xu,2010 | Unclear | Unclear | Unclear | Low Risk | Low Risk | Unclear |
| Xiong,2010 | Unclear | Unclear | Unclear | Low Risk | Low Risk | Unclear |
| Long,2011 | Unclear | Unclear | Unclear | Low Risk | Low Risk | Unclear |
| Jin,2011 | Unclear | Unclear | Unclear | Low Risk | Low Risk | Unclear |
| Ren,2012 | Low Risk | Unclear | Unclear | Low Risk | Low Risk | Unclear |
| Sun,2012 | Unclear | Unclear | Unclear | Low Risk | Low Risk | Unclear |
| Wu,2012 | Unclear | Unclear | Unclear | Low Risk | Low Risk | Unclear |
| Li,2012 | Unclear | Unclear | Unclear | Low Risk | Low Risk | Unclear |
| Huang,2013 | Low Risk | Low Risk | Low Risk | Low Risk | Low Risk | Unclear |
| Dai,2013 | Low Risk | Unclear | Unclear | Low Risk | Low Risk | Unclear |
| Shao,2013 | Unclear | Unclear | Unclear | Low Risk | Low Risk | Unclear |
| Li,2014 | Unclear | Unclear | Unclear | Low Risk | Low Risk | Unclear |
| Wang,2014 | Unclear | Unclear | Unclear | Low Risk | Low Risk | Unclear |
| Wang,2015 | Unclear | Unclear | Unclear | Low Risk | Low Risk | Unclear |
| Wu,2015 | Unclear | Unclear | Unclear | Low Risk | Low Risk | Unclear |
| Ho,2015 | Low Risk | Unclear | Unclear | Low Risk | Low Risk | Unclear |
| Hong,2015 | Unclear | Unclear | Unclear | Low Risk | Low Risk | Unclear |
| Wang,2015 | Unclear | Unclear | Unclear | Low Risk | Low Risk | Unclear |
| Cai,2015 | Unclear | Unclear | Unclear | Low Risk | Low Risk | Unclear |
| Gong,2016 | Unclear | Unclear | Unclear | Low Risk | Low Risk | Unclear |
| Ren,2016 | Unclear | Unclear | Unclear | Low Risk | Low Risk | Unclear |
| Yang,2016 | Low Risk | Unclear | Unclear | Low Risk | Low Risk | Unclear |
| Chen,2017 | Low Risk | Unclear | Unclear | Low Risk | Low Risk | Unclear |
| Buti,2017 | Low Risk | Low Risk | Unclear | Low Risk | Low Risk | Unclear |
| Fan,2017 | Unclear | Unclear | Unclear | Low Risk | Low Risk | Unclear |
| Luo,2017 | Low Risk | Unclear | Unclear | Low Risk | Low Risk | Unclear |
| Hu,2017 | Unclear | Unclear | Unclear | Low Risk | Low Risk | Unclear |
| Li,2017 | Low Risk | Unclear | Unclear | Low Risk | Low Risk | Unclear |
| Zhan,2018 | Unclear | Unclear | Unclear | Low Risk | Low Risk | Unclear |
| Zhang,2018 | Low Risk | Unclear | Unclear | Low Risk | Low Risk | Unclear |
| Huang,2018 | Low Risk | Unclear | Unclear | Low Risk | Low Risk | Unclear |
| Zheng,2018 | Low Risk | Unclear | Unclear | Low Risk | Low Risk | Unclear |
| Wang,2018 | Low Risk | Unclear | Unclear | Low Risk | Low Risk | Unclear |
| Zhang,2018 | Low Risk | Unclear | Unclear | Low Risk | Low Risk | Unclear |
| Li,2019 | Low Risk | Unclear | Unclear | Low Risk | Low Risk | Unclear |
| Liu,2019 | Low Risk | Unclear | Unclear | Low Risk | Low Risk | Unclear |
| Chen,2019 | Unclear | Unclear | Unclear | Low Risk | Low Risk | Unclear |
| Qiu,2019 | Low Risk | Unclear | Unclear | Low Risk | Low Risk | Unclear |
| Toka,2020 | Low Risk | Unclear | Unclear | Low Risk | Low Risk | Unclear |

Table S3 1-year survival rate

| First Author, Year | Randomization | Blinding | Allocation concealment | Incomplete outcome data | Intent to treat analysis | Other sources of bias |
| --- | --- | --- | --- | --- | --- | --- |
| Xu,2009 | Unclear | Unclear | Unclear | Low Risk | Low Risk | Unclear |
| Zhan,2010 | Unclear | Unclear | Unclear | Low Risk | Low Risk | Unclear |
| Fang,2011 | Unclear | Unclear | Unclear | Low Risk | Low Risk | Unclear |
| Fang,2012 | Unclear | Unclear | Unclear | Low Risk | Low Risk | Unclear |
| Shao,2013 | Unclear | Unclear | Unclear | Low Risk | Low Risk | Unclear |
| Dai,2013 | Unclear | Unclear | Unclear | Low Risk | Low Risk | Unclear |
| Zhang,2013 | Unclear | Unclear | Unclear | Low Risk | Low Risk | Unclear |
| Li,2014 | Unclear | Unclear | Unclear | Low Risk | Low Risk | Unclear |
| Wan,2014 | Low Risk | Unclear | Unclear | Low Risk | Low Risk | Unclear |
| Wang,2014 | Unclear | Unclear | Unclear | Low Risk | Low Risk | Unclear |
| Xu,2014 | Unclear | Unclear | Unclear | Low Risk | Low Risk | Unclear |
| Xu,2014 | Unclear | Unclear | Unclear | Low Risk | Low Risk | Unclear |
| Huang,2015 | Low Risk | Unclear | Unclear | Low Risk | Low Risk | Unclear |
| Wang,2015 | Unclear | Unclear | Unclear | Low Risk | Low Risk | Unclear |
| Pan,2015 | Unclear | Unclear | Unclear | Low Risk | Low Risk | Unclear |
| Bai,2016 | Low Risk | Unclear | Unclear | Low Risk | Low Risk | Unclear |
| Yin,2016 | Unclear | Unclear | Unclear | Low Risk | Low Risk | Unclear |
| Yang,2016 | Low Risk | Unclear | Unclear | Low Risk | Low Risk | Unclear |
| Hu,2017 | Low Risk | Unclear | Unclear | Low Risk | Low Risk | Unclear |
| Jiang,2017 | Unclear | Unclear | Unclear | Low Risk | Low Risk | Unclear |
| Li,2017 | Low Risk | Unclear | Unclear | Low Risk | Low Risk | Unclear |
| Tang,2018 | Low Risk | Unclear | Unclear | Low Risk | Low Risk | Unclear |
| Huang,2018 | Low Risk | Unclear | Unclear | Low Risk | Low Risk | Unclear |
| Lu,2019 | Low Risk | Unclear | Unclear | Low Risk | Low Risk | Unclear |
| Gao,2020 | Low Risk | Unclear | Unclear | Low Risk | Low Risk | Unclear |

Table S4 2-year survival rate

| First Author, Year | Randomization | Blinding | Allocation concealment | Incomplete outcome data | Intent to treat analysis | Other sources of bias |
| --- | --- | --- | --- | --- | --- | --- |
| Zhan,2010 | Unclear | Unclear | Unclear | Low Risk | Low Risk | Unclear |
| Fang,2011 | Unclear | Unclear | Unclear | Low Risk | Low Risk | Unclear |
| Fang,2012 | Unclear | Unclear | Unclear | Low Risk | Low Risk | Unclear |
| Dai,2013 | Unclear | Unclear | Unclear | Low Risk | Low Risk | Unclear |
| Wang,2014 | Unclear | Unclear | Unclear | Low Risk | Low Risk | Unclear |
| Xu,2014 | Unclear | Unclear | Unclear | Low Risk | Low Risk | Unclear |
| Xu,2014 | Unclear | Unclear | Unclear | Low Risk | Low Risk | Unclear |
| Hou,2015 | Unclear | Unclear | Unclear | Unclear | Unclear | Unclear |
| Pan,2015 | Unclear | Unclear | Unclear | Low Risk | Low Risk | Unclear |
| Bai,2016 | Low Risk | Unclear | Unclear | Low Risk | Low Risk | Unclear |
| Yang,2016 | Low Risk | Unclear | Unclear | Low Risk | Low Risk | Unclear |
| Hu,2017 | Low Risk | Unclear | Unclear | Low Risk | Low Risk | Unclear |
| Hu,2017 | Unclear | Unclear | Unclear | Low Risk | Low Risk | Unclear |
| Liang,2018 | Unclear | Unclear | Unclear | Low Risk | Low Risk | Unclear |
| Lu,2019 | Low Risk | Unclear | Unclear | Low Risk | Low Risk | Unclear |
| Yang,2019 | Low Risk | Unclear | Unclear | Low Risk | Low Risk | Unclear |
| Gao,2020 | Low Risk | Unclear | Unclear | Low Risk | Low Risk | Unclear |

Table S5 3-year survival rate

| First Author, Year | Randomization | Blinding | Allocation concealment | Incomplete outcome data | Intent to treat analysis | Other sources of bias |
| --- | --- | --- | --- | --- | --- | --- |
| Zhang,2010 | Unclear | Unclear | Unclear | Low Risk | Low Risk | Unclear |
| Fang,2012 | Unclear | Unclear | Unclear | Low Risk | Low Risk | Unclear |
| Xu,2014 | Unclear | Unclear | Unclear | Low Risk | Low Risk | Unclear |
| Huang,2015 | Low Risk | Unclear | Unclear | Low Risk | Low Risk | Unclear |
| Pan,2015 | Unclear | Unclear | Unclear | Low Risk | Low Risk | Unclear |
| Bai,2016 | Low Risk | Unclear | Unclear | Low Risk | Low Risk | Unclear |
| Hu,2017 | Low Risk | Unclear | Unclear | Low Risk | Low Risk | Unclear |
| Liang,2018 | Unclear | Unclear | Unclear | Low Risk | Low Risk | Unclear |
| Huang,2018 | Low Risk | Unclear | Unclear | Low Risk | Low Risk | Unclear |
| Tang,2018 | Low Risk | Unclear | Unclear | Low Risk | Low Risk | Unclear |

Table S6 Chemotherapy disruption rate

| First Author, Year | Randomization | Blinding | Allocation concealment | Incomplete outcome data | Intent to treat analysis | Other sources of bias |
| --- | --- | --- | --- | --- | --- | --- |
| Zheng,2018 | Low Risk | Unclear | Unclear | Low Risk | Low Risk | Unclear |
| Jang,2006 | Low Risk | Unclear | Unclear | Low Risk | Low Risk | Unclear |
| Zeng,2007 | Unclear | Unclear | Unclear | Low Risk | Low Risk | Unclear |
| Duan,2009 | Unclear | Unclear | Unclear | Low Risk | Low Risk | Unclear |
| Xiong,2010 | Unclear | Unclear | Unclear | Low Risk | Low Risk | Unclear |
| Jin,2011 | Unclear | Unclear | Unclear | Low Risk | Low Risk | Unclear |
| Wu,2012 | Unclear | Unclear | Unclear | Low Risk | Low Risk | Unclear |
| Ren,2012 | Low Risk | Unclear | Unclear | Low Risk | Low Risk | Unclear |
| Wang,2014 | Unclear | Unclear | Unclear | Low Risk | Low Risk | Unclear |
| Yang,2016 | Low Risk | Unclear | Unclear | Low Risk | Low Risk | Unclear |
| Fan,2017 | Unclear | Unclear | Unclear | Low Risk | Low Risk | Unclear |
| Li,2019 | Unclear | Unclear | Unclear | Low Risk | Low Risk | Unclear |
| Zhang,2020 | Low Risk | Unclear | Unclear | Low Risk | Low Risk | Unclear |

# Supplementary Information 4. Inconsistency test table（including global inconsistency、Node-splitting and Inconsistency test for closed loops）

Table S7 Global inconsistency of all indicators

| Outcome indicator | Reactivation rate | 1-year survival rate | 2-year survival rate | Chemotherapy disruption rate |
| --- | --- | --- | --- | --- |
| *P*-value | 0.3492 | 0.5752 | 0.3074 | 0.2397 |

Table S8 node-splitting：

a. Reactivation rate：

| Side | Direct Coef | Std.Err | Indirect Coef | Std.Err | Difference Coef | Std.Err | P valule |
| --- | --- | --- | --- | --- | --- | --- | --- |
| A B | -1.498343 | 0.198094 | -3.196303 | 0.8773559 | 1.69796 | 0.8994413 | 0.059 |
| A C | -1.542015 | 0.170789 | 0.0785956 | 0.8332763 | -1.620611 | 0.860823 | 0.060 |
| A D | -1.491756 | 0.5688516 | -1.685804 | 0.3829686 | 0.1940484 | 0.671825 | 0.773 |
| A E | . | . | . | . | . | . | . |
| A F | -2.104975 | 1.490577 | -1.290365 | 0.6574799 | -0.8146101 | 1.629141 | 0.617 |
| B C | 2.025954 | 1.030887 | -0.0023096 | 0.2580313 | 2.023644 | 1.062689 | 0.057 |
| B F | 0.2787135 | 0.628014 | -0.535893 | 1.503229 | 0.8146065 | 1.62914 | 0.617 |
| C D | -0.1721247 | 0.3218618 | -0.1300876 | 1.027818 | -0.0420371 | 1.097746 | 0.969 |
| C G | -1.317451 | 0.3776701 | 2.512856 | 870.9748 | -3.830306 | 870.9748 | 0.996 |
| C H | -0.3730298 | 0.507964 | 2.74635 | 1133.567 | -3.11938 | 1133.567 | 0.998 |

A=BC、B=ETV、C=LAM、D=ADV、E=LdT、F=TDF、G=LAM+ETV、H=LAM+ADV

b. 1-year survival rate：

| Side | Direct Coef | Std.Err | Indirect Coef | Std.Err | Difference Coef | Std.Err | P valule |
| --- | --- | --- | --- | --- | --- | --- | --- |
| A C | 0.2506523 | 0.0549615 | -0.0610014 | 0.314315 | 0.3116537 | 0.3233488 | 0.335 |
| A D | 0.0587576 | 0.069529 | 0.1336057 | 0.2937604 | -0.074848 | 0.307213 | 0.808 |
| C D | -0.0913044 | 0.1490496 | -0.2062014 | 0.0923602 | 0.114897 | 0.1750112 | 0.511 |

A=BC、B=ETV、C=LAM、D=ADV、E=LdT、F=LAM+ADV（Missing comparison groups because there are no pairwise comparisons for them.）

c. 2-year survival rate：

| Side | Direct Coef | Std.Err | Indirect Coef | Std.Err | Difference Coef | Std.Err | P valule |
| --- | --- | --- | --- | --- | --- | --- | --- |
| A C | 0.3019767 | 0.0894262 | -0.1691891 | 0.5916483 | 0.4711658 | 0.6064334 | 0.437 |
| A D | 0.38527 | 0.1774514 | 0.0923161 | 0.4480281 | 0.2929538 | 0.5163044 | 0.570 |
| C D | 0.0545153 | 0.2123859 | 0.0343192 | 0.2262672 | 0.0201961 | 0.3118055 | 0.948 |

A=BC、B=ETV、C=LAM、D=ADV（Missing comparison groups because there are no pairwise comparisons for them.）

d. Chemotherapy disruption rate：

| Side | Direct Coef | Std.Err | Indirect Coef | Std.Err | Difference Coef | Std.Err | P valule |
| --- | --- | --- | --- | --- | --- | --- | --- |
| A B | -1.455415 | 0.4755041 | -2.382524 | 0.6290016 | 0.9271093 | 0.7885094 | 0.240 |
| A C | -1.203879 | 0.176998 | -0.2767553 | 0.763872 | -0.9271235 | 0.7885095 | 0.240 |
| B C | 1.178655 | .06035961 | 0.2515323 | 0.5073775 | 0.9271231 | 0.7885101 | 0.240 |
| B D | -0.1718503 | 0.244082 | 3.576413 | 667.0178 | -3.748263 | 667.0178 | 0.996 |

A=BC、B=ETV、C=LAM、D=TDF（Missing comparison groups because there are no pairwise comparisons for them.）

Table S9 Inconsistency test for closed loops：Evaluation of loop specific coherence results presenting incoherence factors and 95% confidence intervals (truncated to zero): reactivation rate、survival rate、chemotherapy disruption rate outcomes. Confidence intervals for incoherence factors that include zero are considered to not have concerns about local incoherence.

| reactivation rate | | | |
| --- | --- | --- | --- |
| Closed loop with trial data available | Incoherence Factor (IF) | IF 95% CI | P value |
| BC-ETV-LAM | 2.060 | （0.00,4.14） | 0.053 |
| BC-ETV-TDF | 0.885 | （0.00,4.08） | 0.587 |
| BC-LAM-ADV | 0.303 | （0.00,1.67） | 0.664 |
| 1-year survival rate | | | |
| BC-LAM-ADV | 0.129 | （0.00,0.41） | 0.366 |
| 2-year survival rate | | | |
| BC-LAM-ADV | 0.079 | （0.00,0.57） | 0.752 |
| chemotherapy disruption rate | | | |
| BC-ETV-LAM | 0.927 | （0.00,2.47） | 0.240 |

Table S10 Meta-regression analysis of reactivation rate

| LogRR | Coef | Std.Err | t | P＞│t│ | [95%Conf.Interval] | |
| --- | --- | --- | --- | --- | --- | --- |
| tumor types | -0.0380797 | 0.0399174 | -0.98 | 0.346 | -0.1188202 | 0.0426608 |
| comparison | -0.0242323 | 0.0330113 | -0.73 | 0.467 | -0.0910039 | 0.0425393 |
| follow-up time | 0.0580891 | 0.1638676 | 0.35 | 0.725 | -0.2733644 | 0.3895426 |

# Supplementary Information 5. Heterogeneity test graph


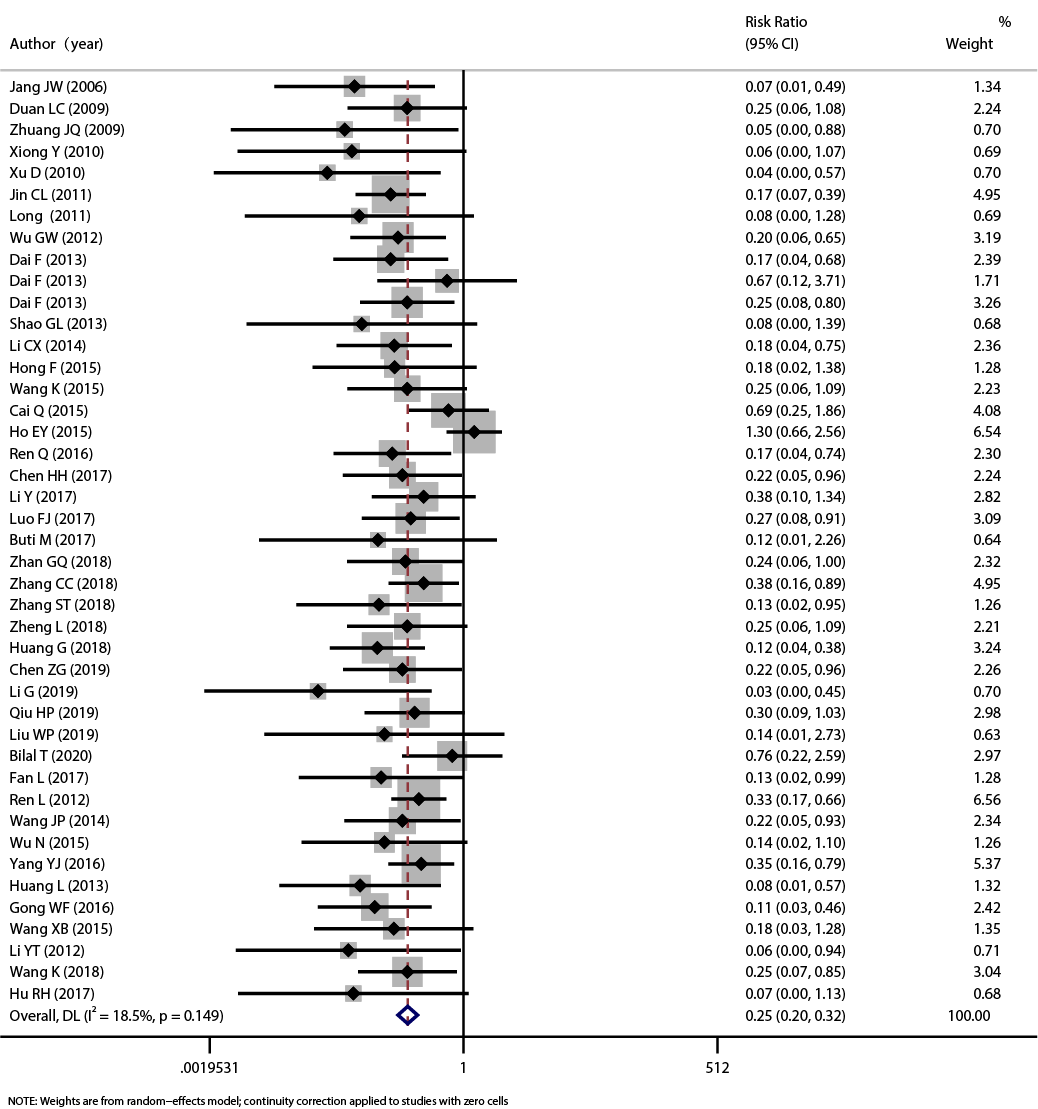
Figure S1 Forest map of direct comparison of antiviral drugs of reactivation rate

|  |
| --- |


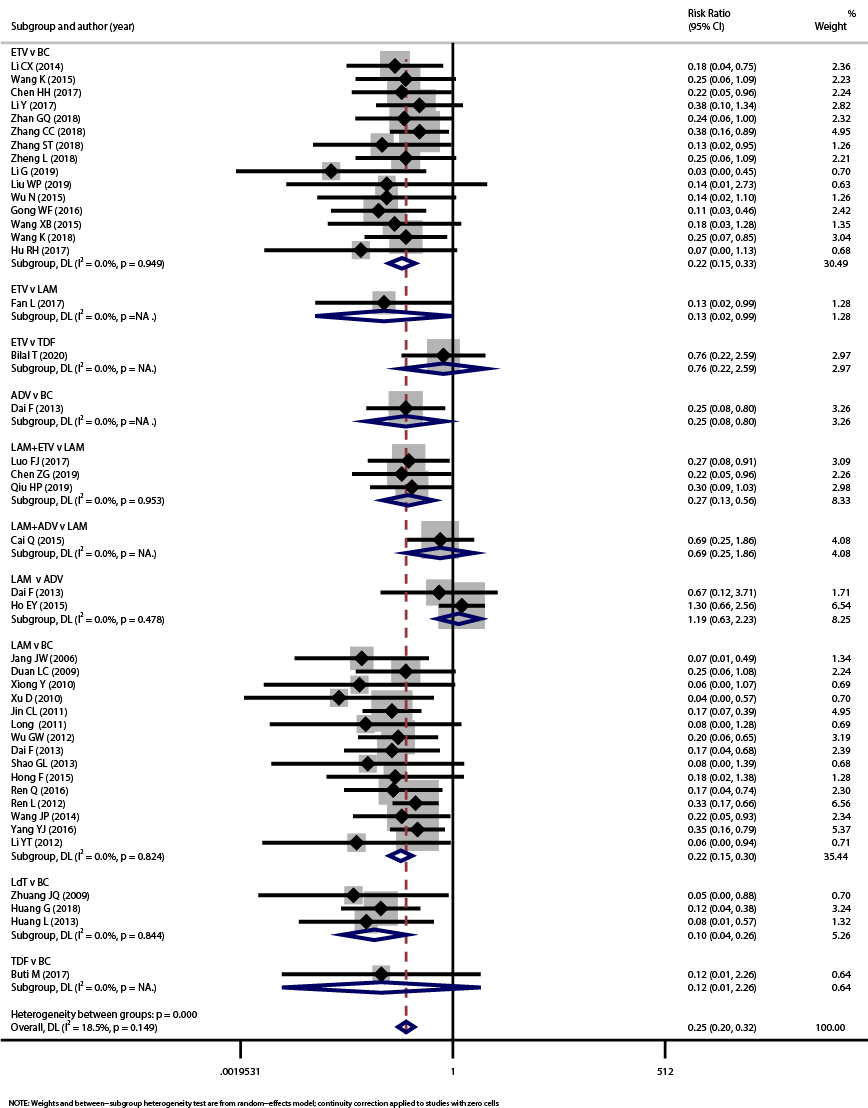
Figure S2 Forest plot of reactivation rates by treatment subgroups

|  |
| --- |

Figure S3 Sensitivity analysis of reactivation rate

| 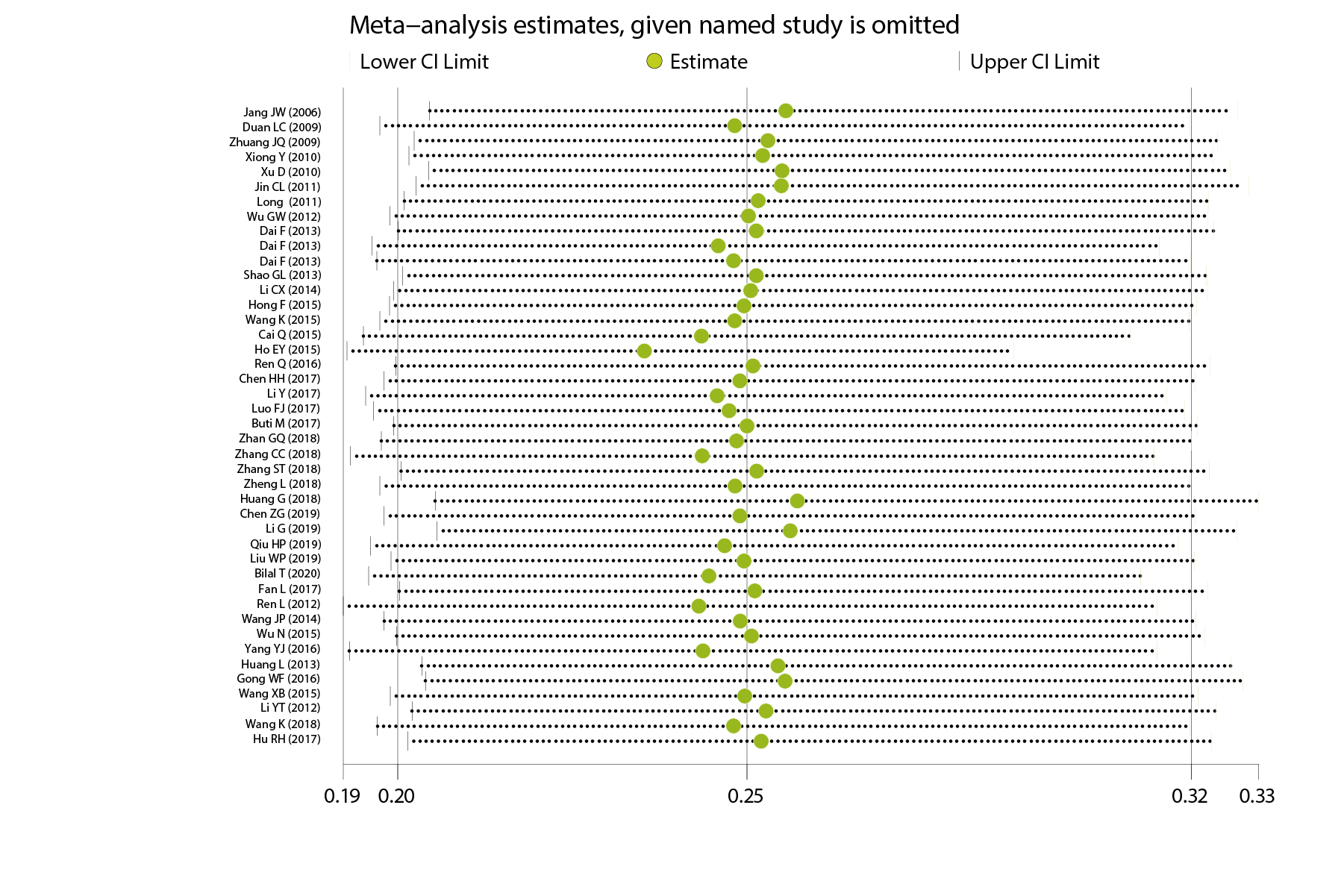 |
| --- |


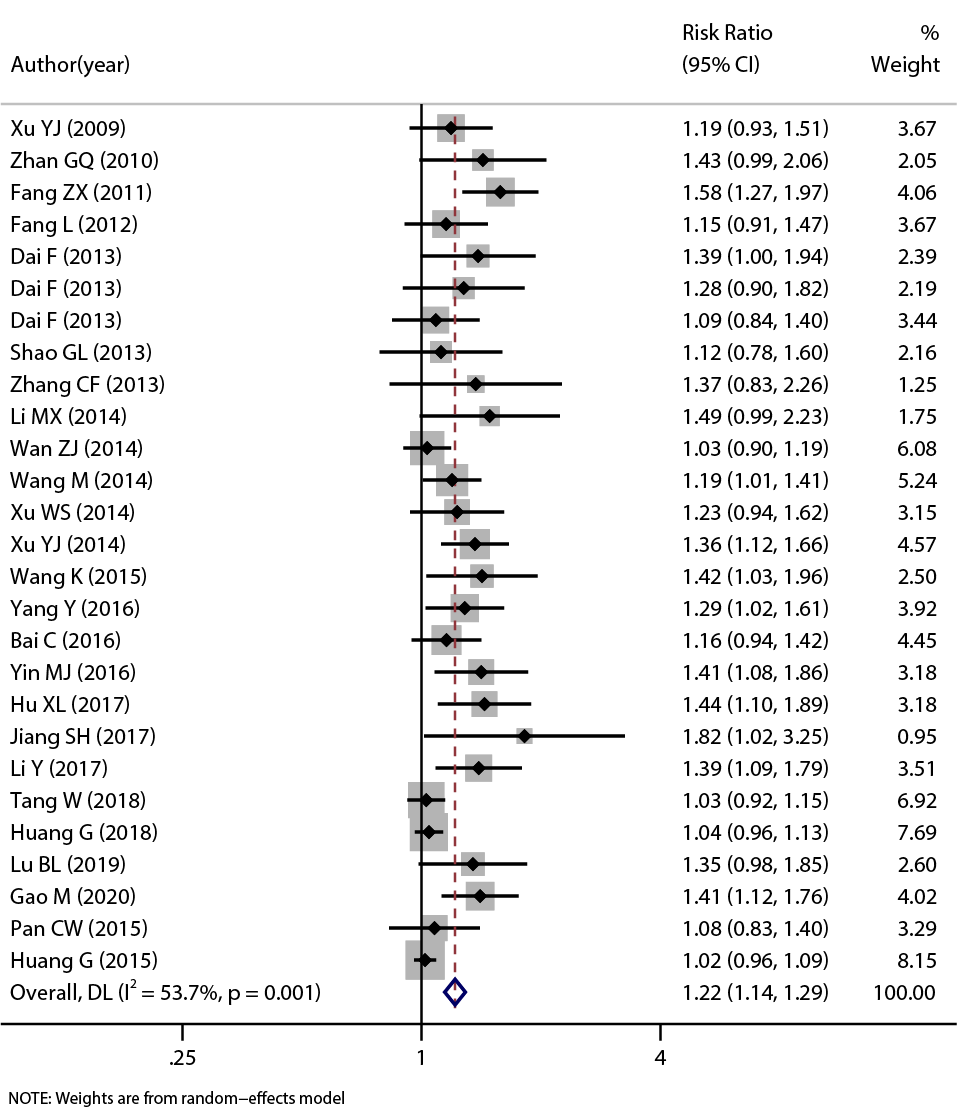
Figure S4 Forest map for direct comparison of 1-year survival rate

|  |
| --- |

Figure S5 Forest plot of 1-year survival rate by treatment subgroups


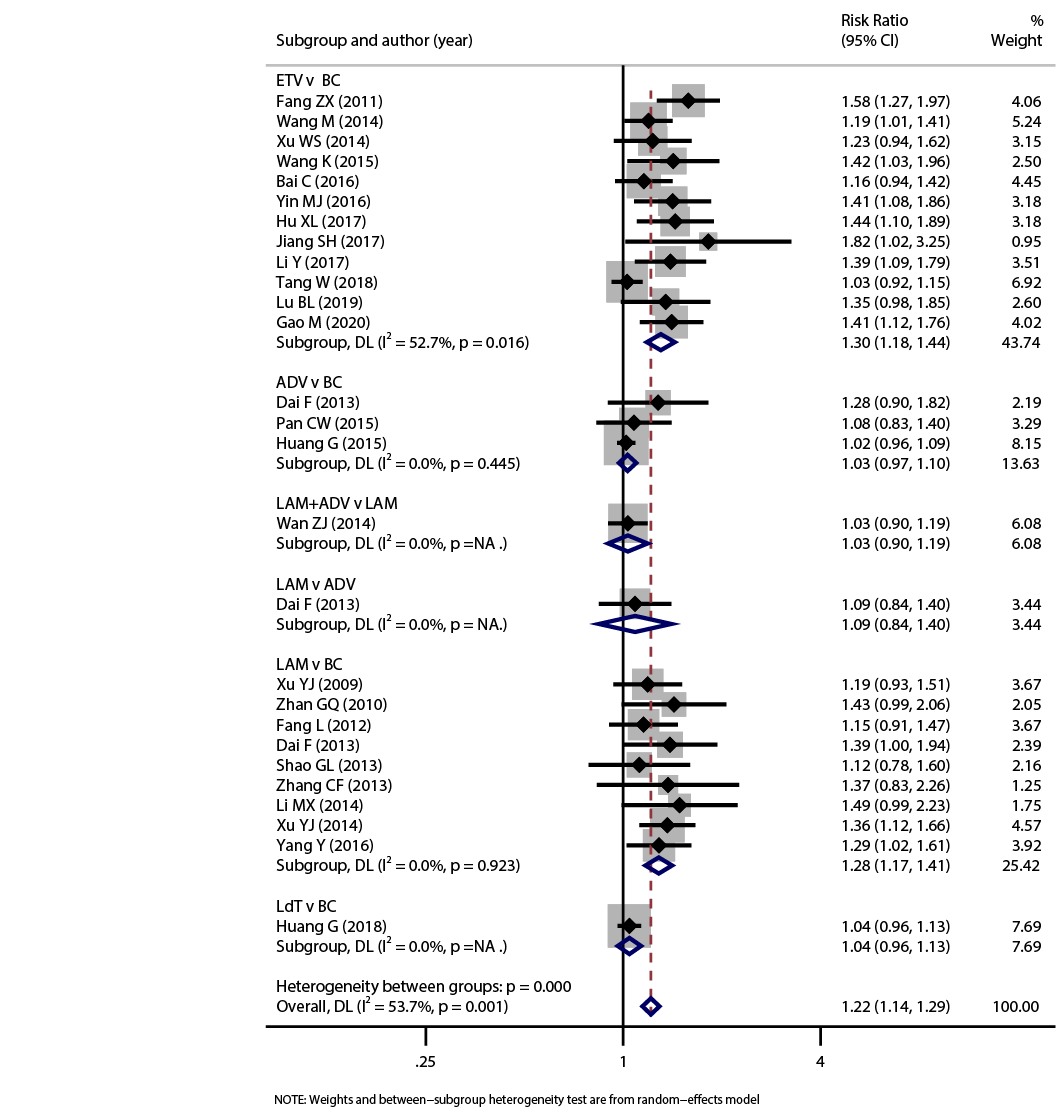


Figure S6 Sensitivity analysis of 1-year survival rate


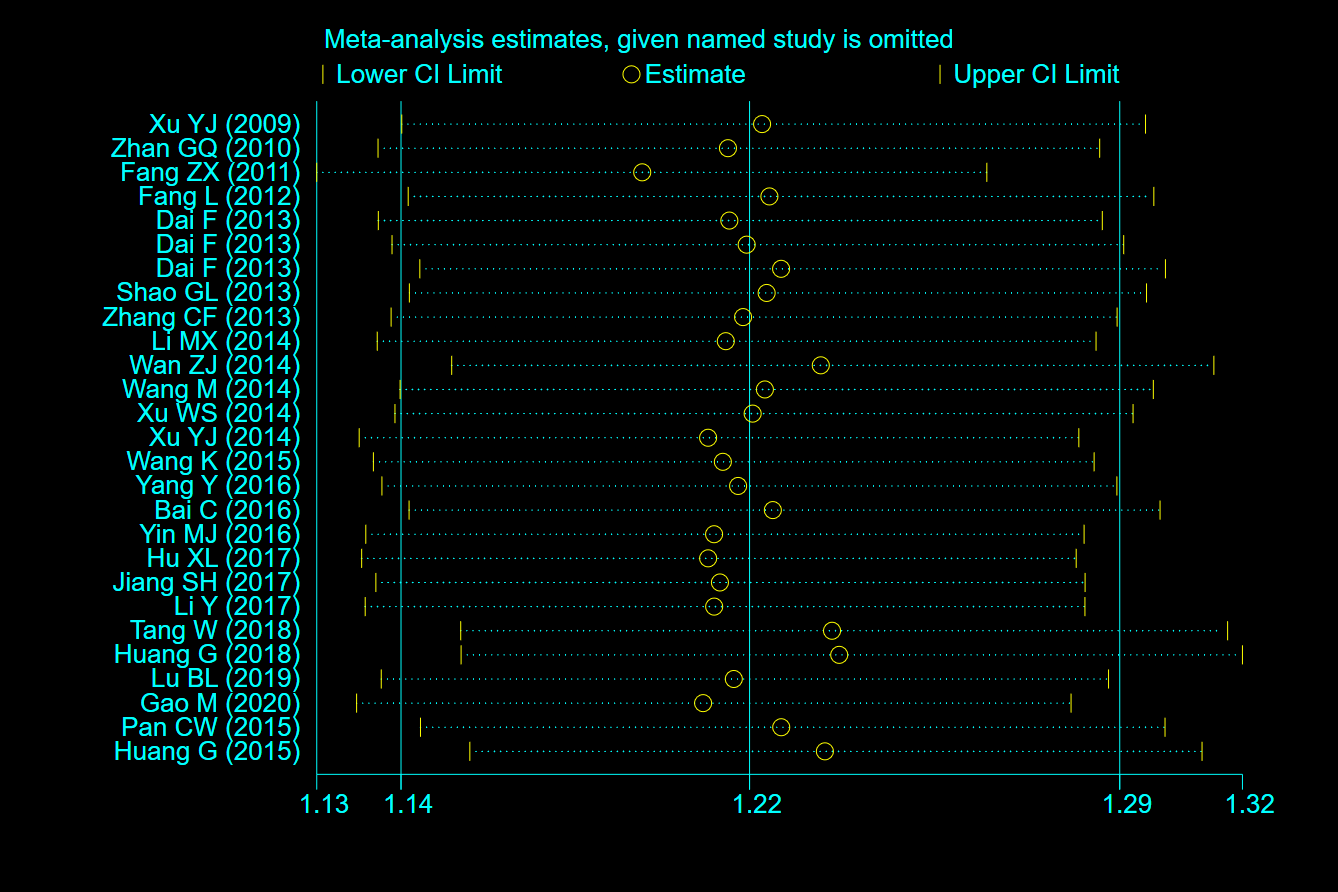


Figure S7 Forest map for direct comparison of 2-year survival rate


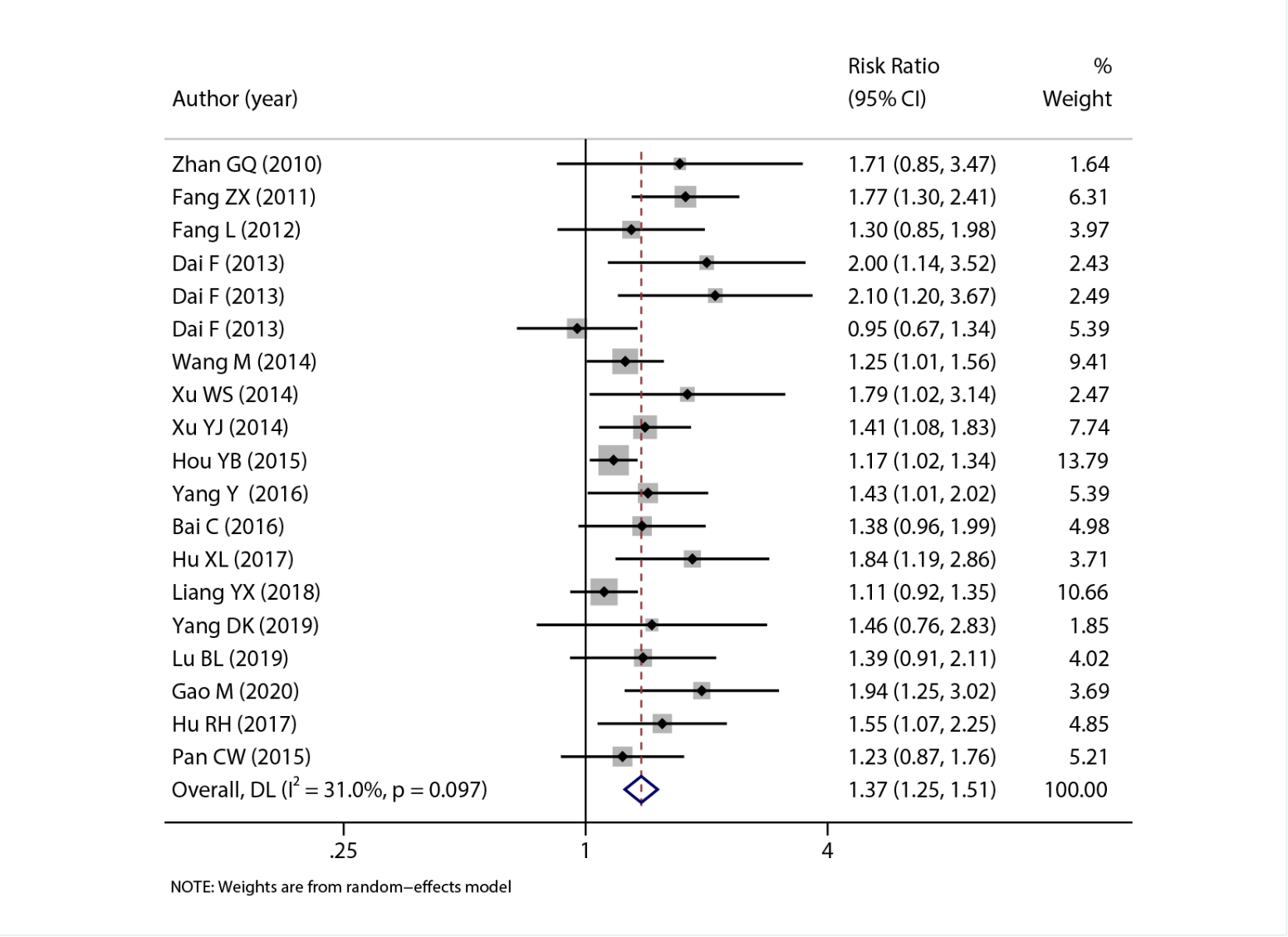


Figure S8 Sensitivity analysis of 2-year survival rate


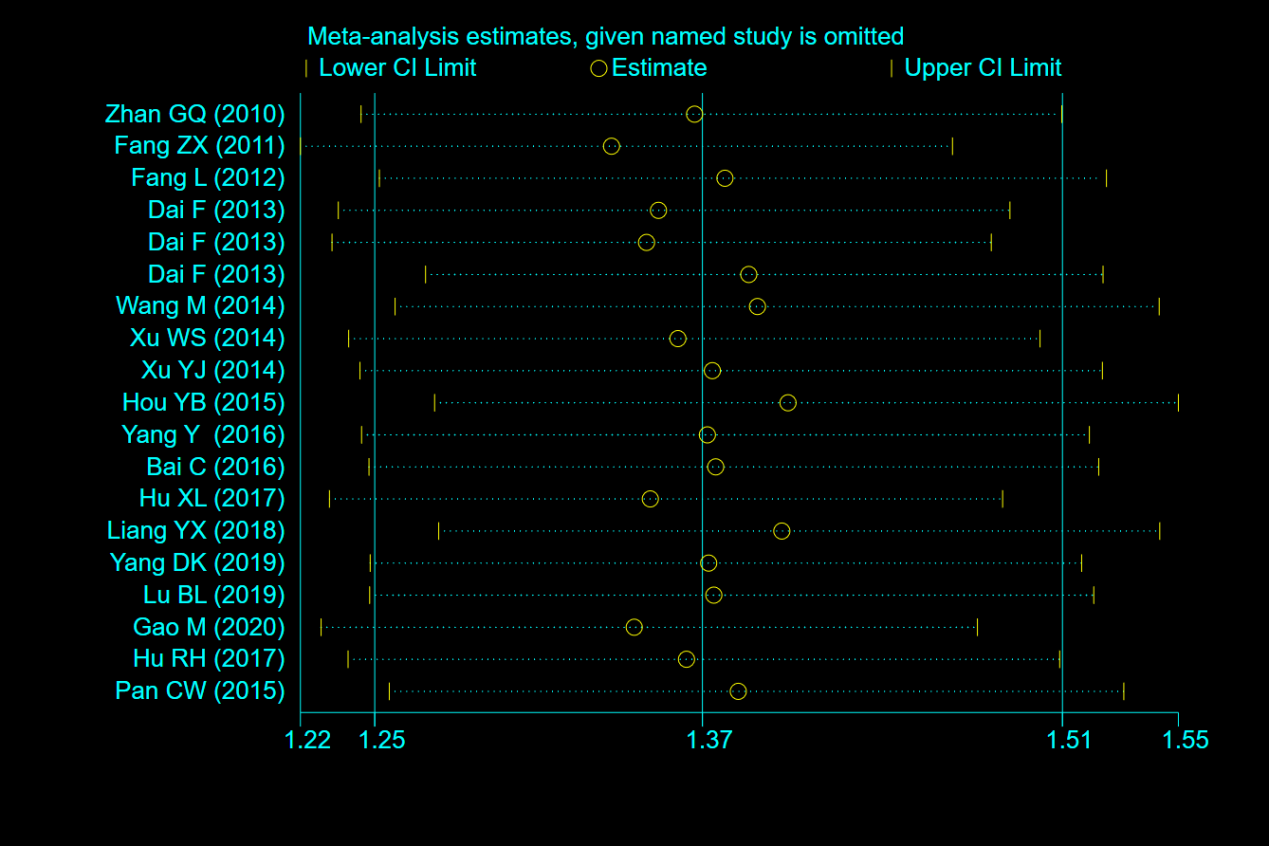


Figure S9 Forest map for direct comparison of 3-year survival rate


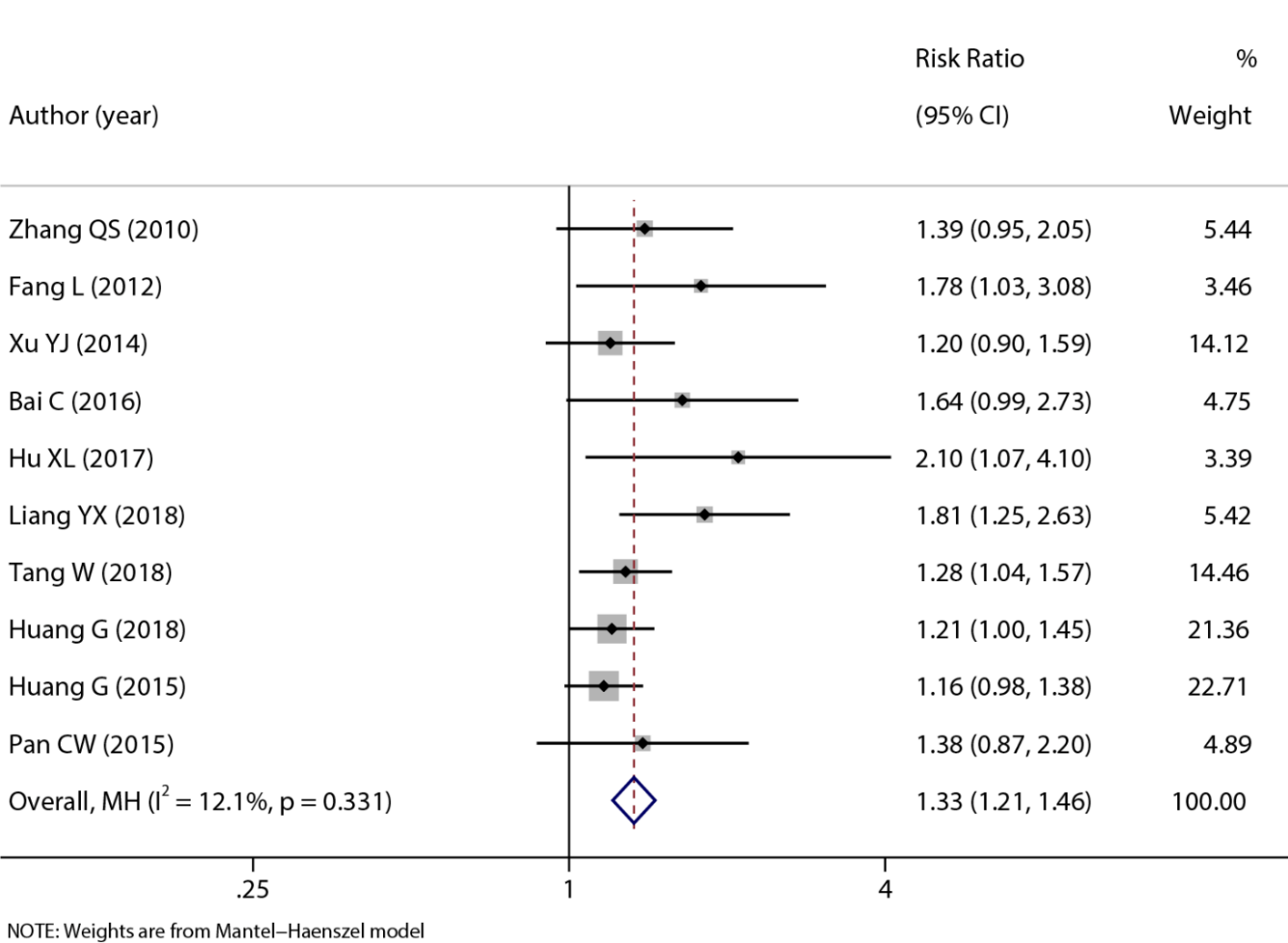


Figure S10 Sensitivity analysis of 3-year survival rate


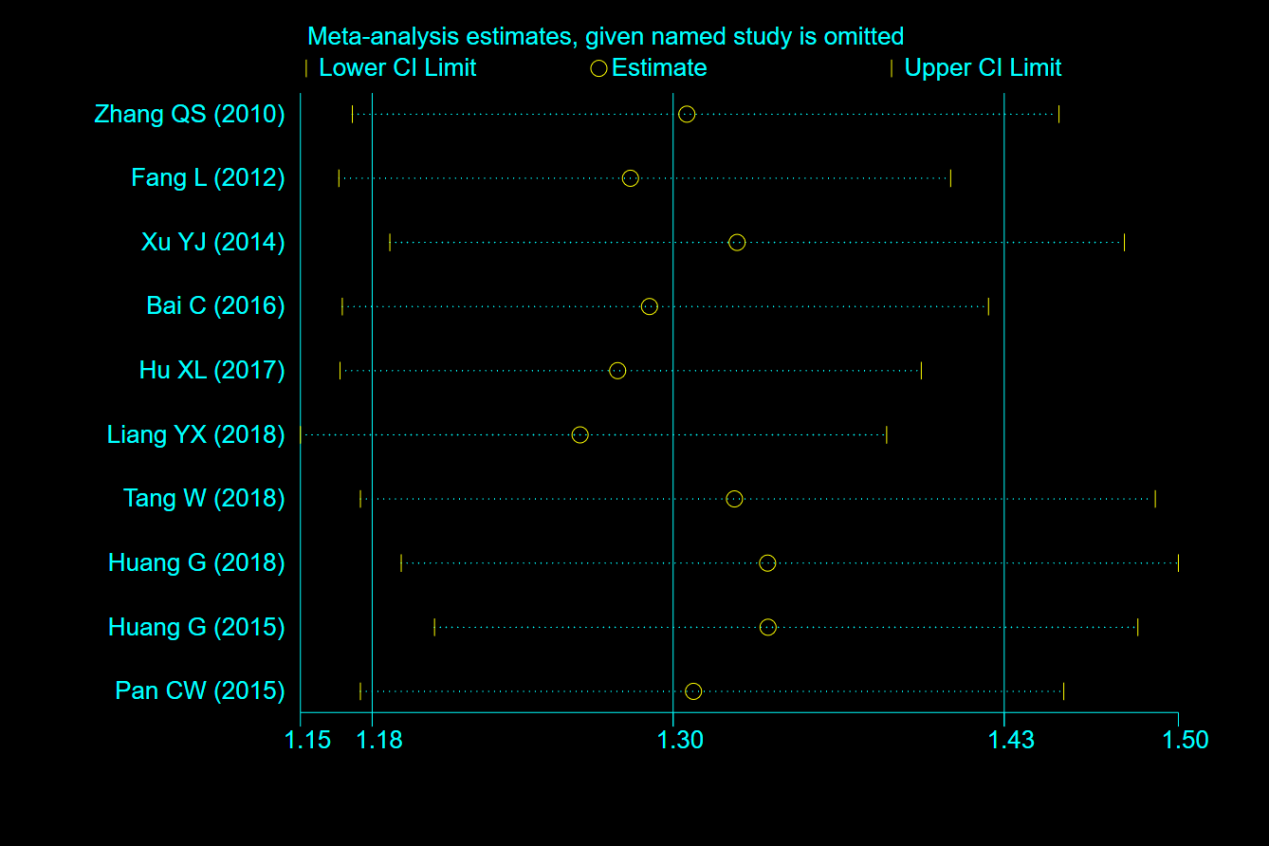


Figure S11 Forest map for direct comparison of chemotherapy disruption rate

| 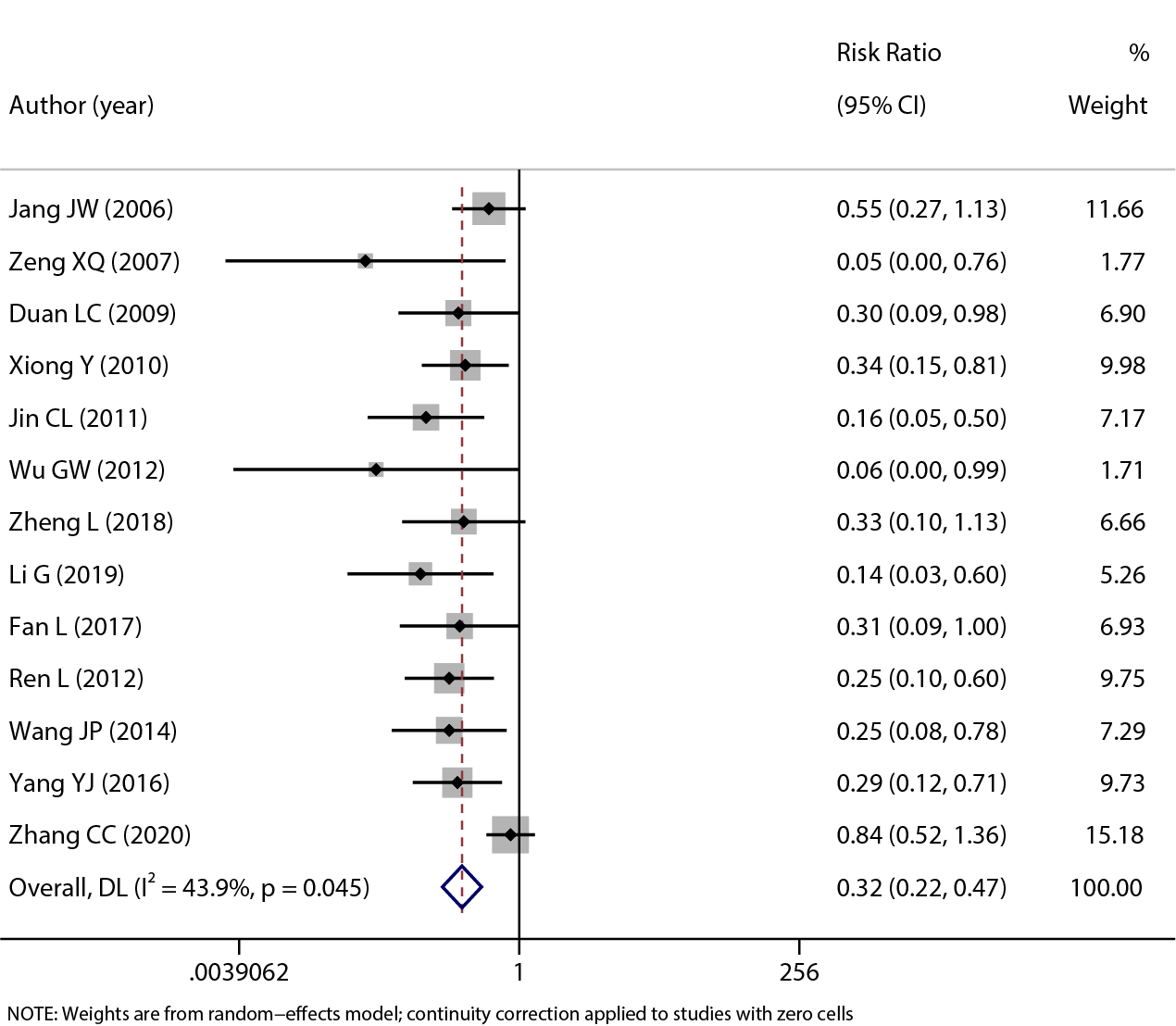 |
| --- |

Figure S12 Sensitivity analysis of chemotherapy disruption rate


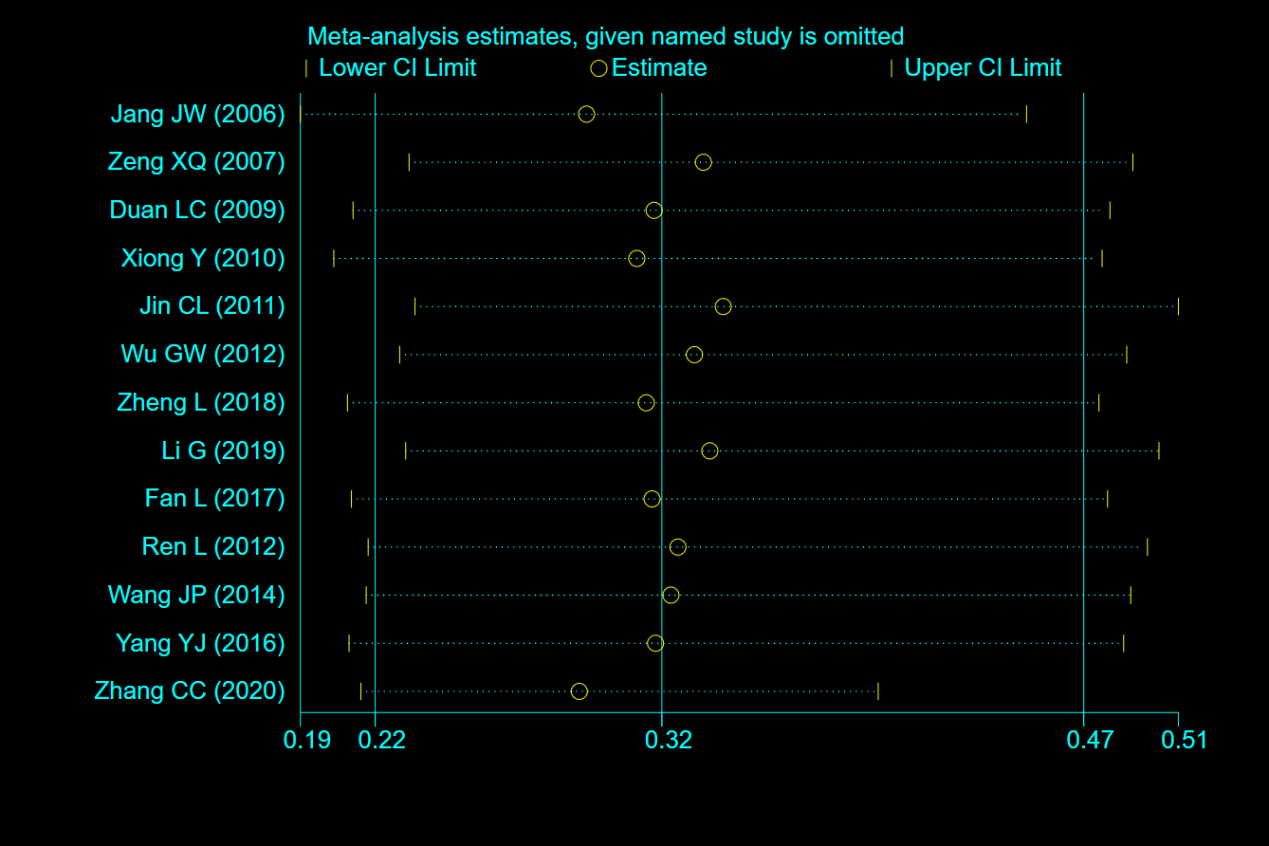


Figure S13A the funnel plot for HBV reactivation rate


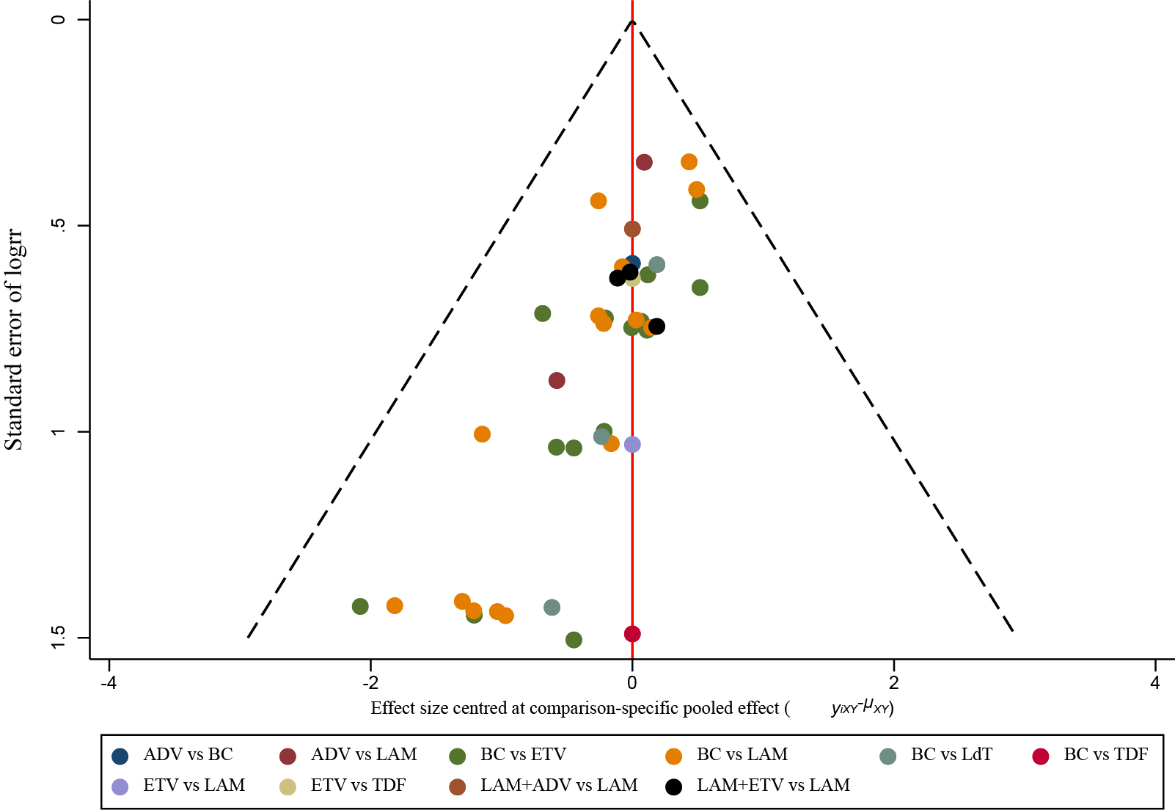


Egger’s p＜0.05

Figure S13B the funnel plot for 1-year survival rate


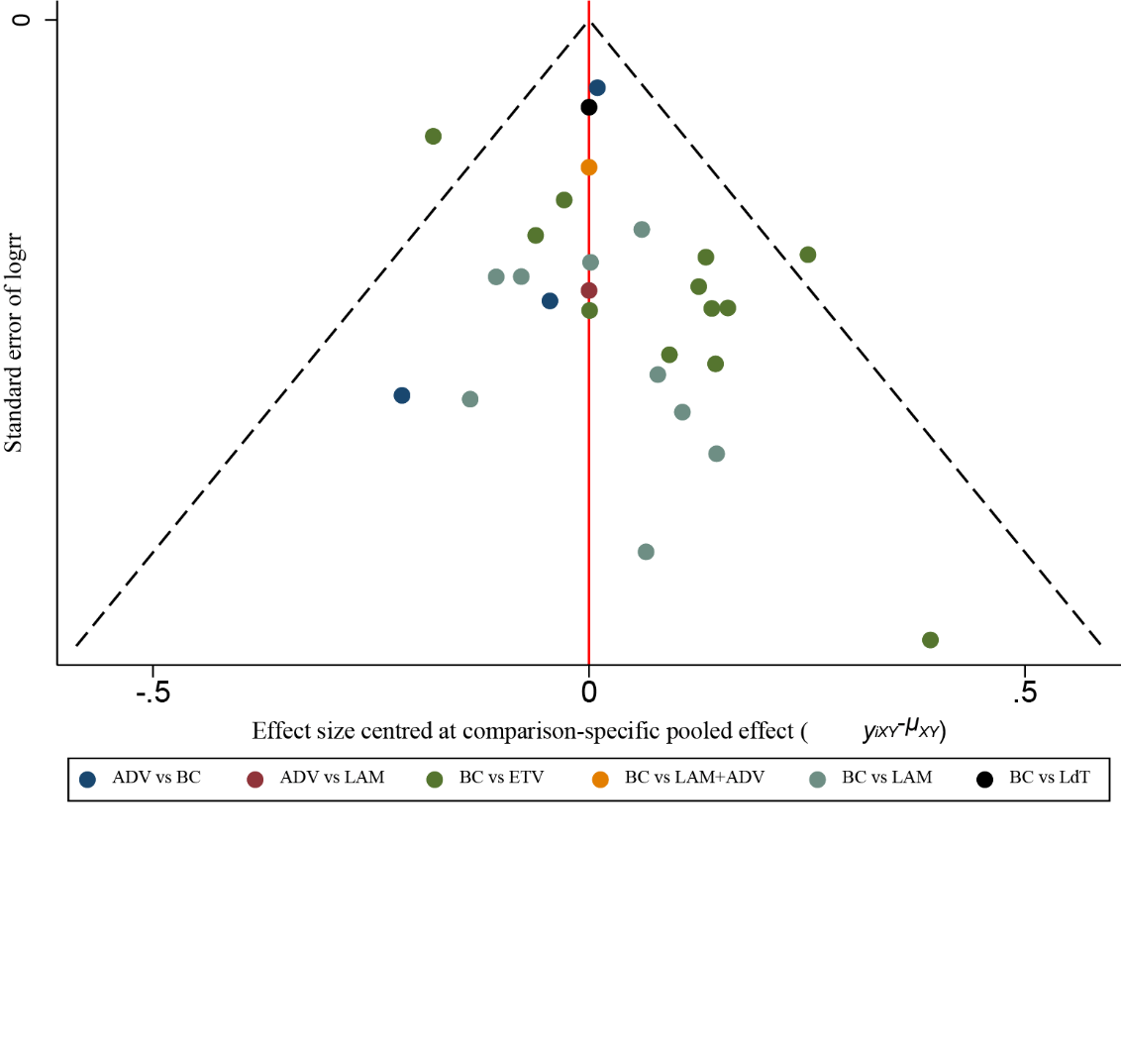


Egger’s p＜0.05

Figure S13C the funnel plot for 2-year survival rate


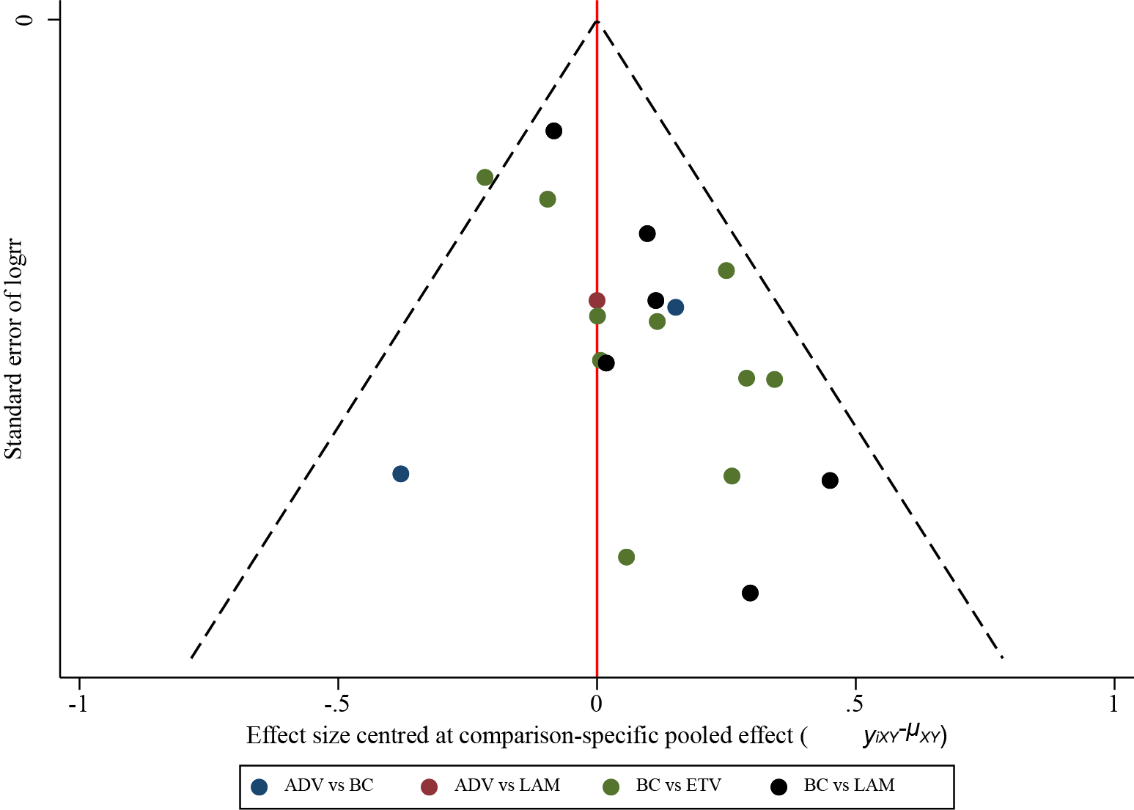


Egger’s p＜0.05

Figure S13D the funnel plot for 3-year survival rate


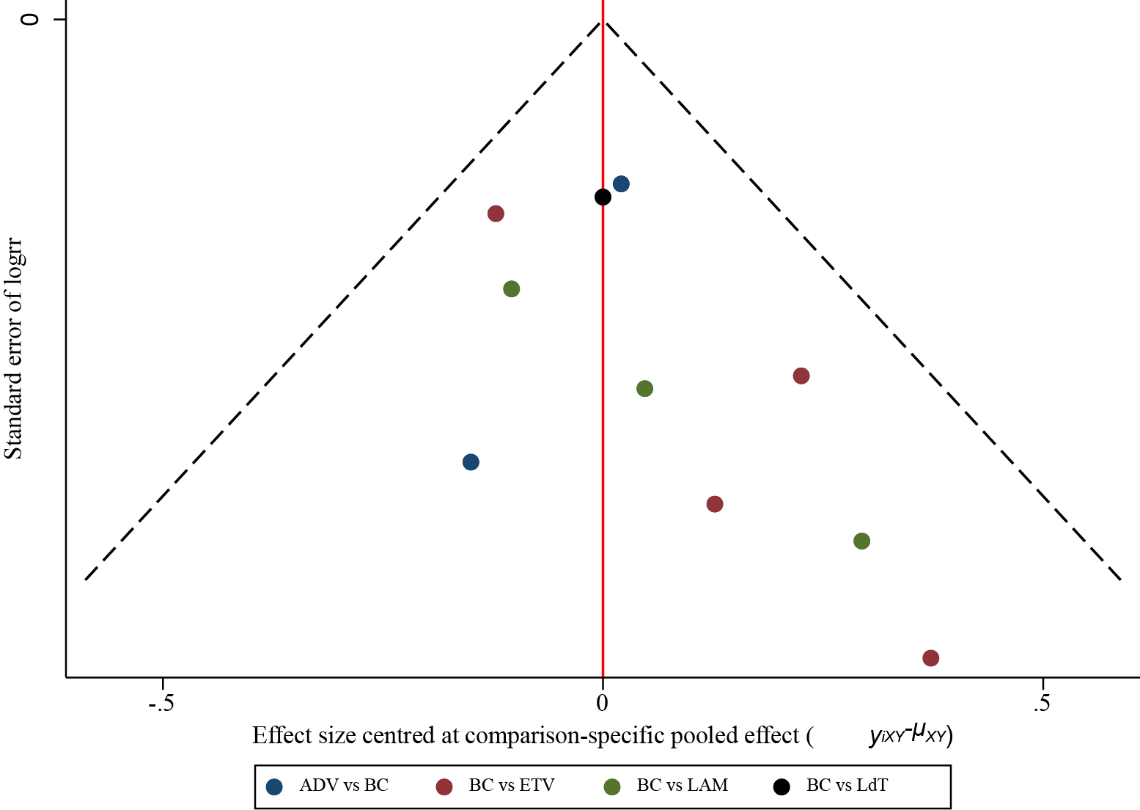


Egger’s p＜0.05

Figure S13E the funnel plot for chemotherapy disruption rate


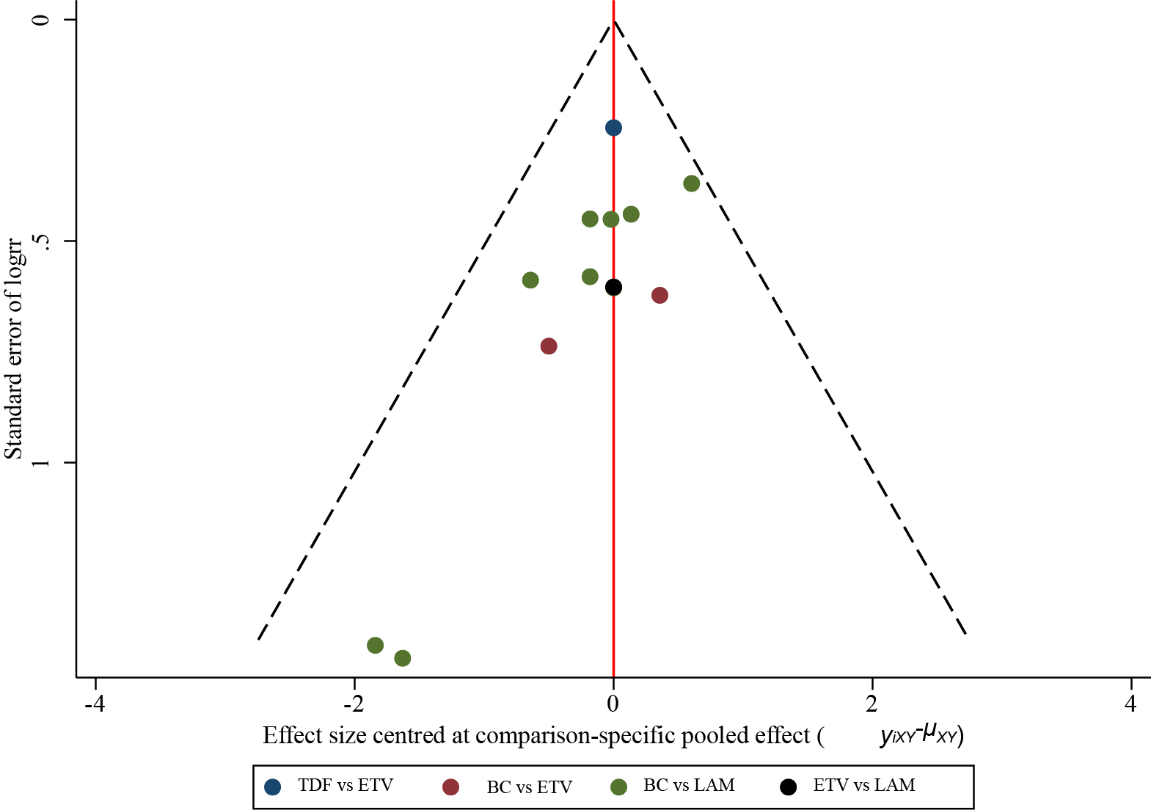


Egger’s p＜0.05

Table S11 Results of the cut-and-patch method for each outcome indicator

| indicator | model | before | | | after | | |
| --- | --- | --- | --- | --- | --- | --- | --- |
|  |  | Pooled Est | 95%CI | P value | Pooled Est | 95%CI | P value |
| reactivation rate | Fixed | -1.282 | -1.487,-1.076 | 0.000 | -1.282 | -1.487,-1.076 | 0.000 |
| 1-year survival rate | Random | 0.194 | 0.134,0.254 | 0.000 | 0.083 | 0.019,0.146 | 0.000 |
| 2-year survival rate | Fixed | 0.278 | 0.208,0.349 | 0.000 | 0.203 | 0.138,0.267 | 0.000 |
| 3-year survival rate | Fixed | 0.254 | 0.165,0.343 | 0.000 | 0.203 | 0.120,0.287 | 0.000 |
| Chemotherapy disruption rate | Fixed | -0.913 | -1.175,-0.650 | 0.000 | -0.913 | -1.175,-0.650 | 0.000 |

Figure S14 Cut-and-patch method illustration for HBV reactivation rate


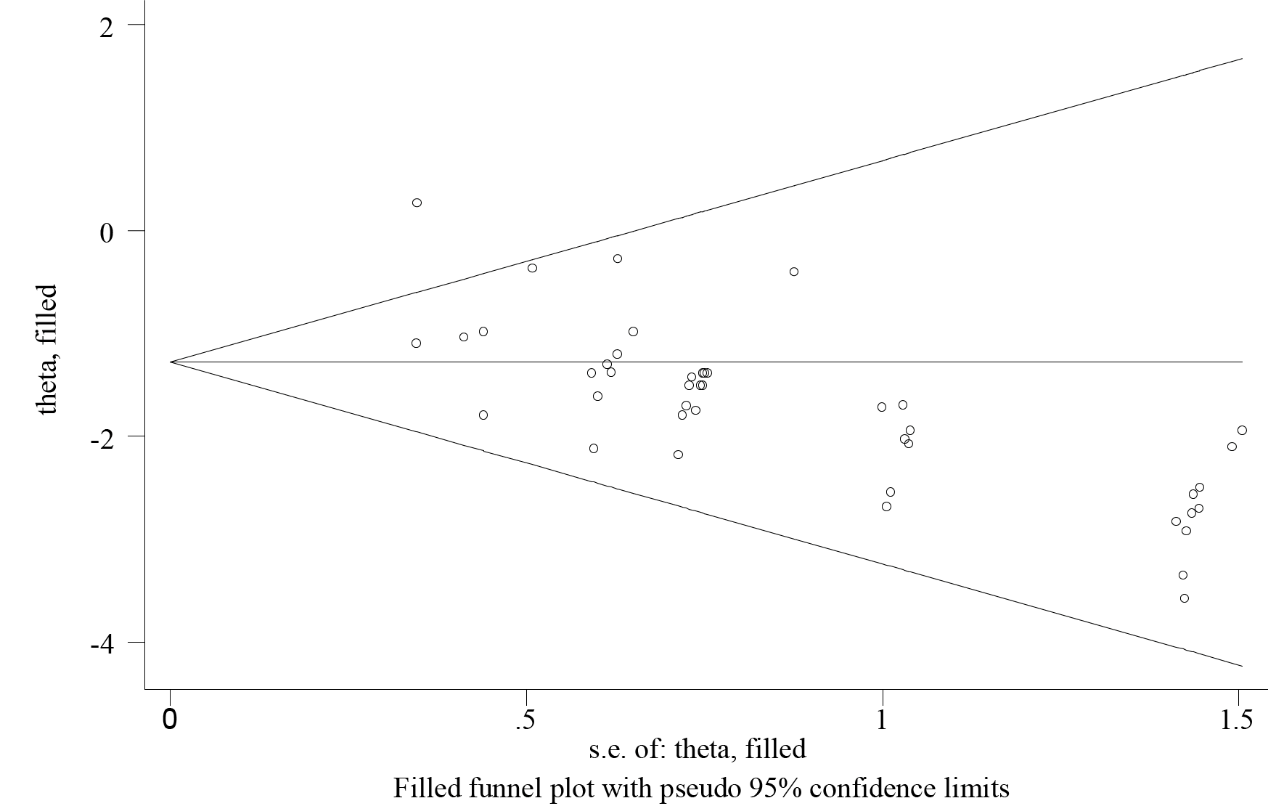


Figure S14 Cut-and-patch method illustration for 1-year survival rate


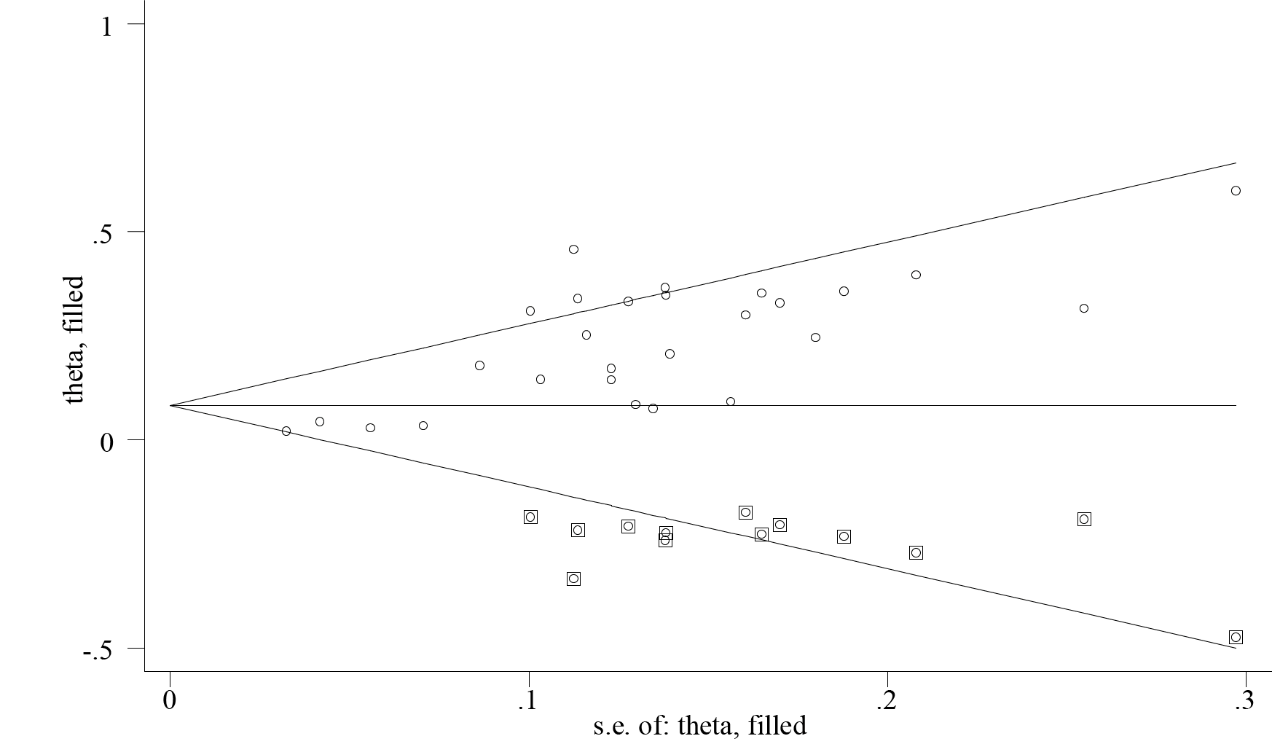


Figure S14 Cut-and-patch method illustration for 2-year survival rate


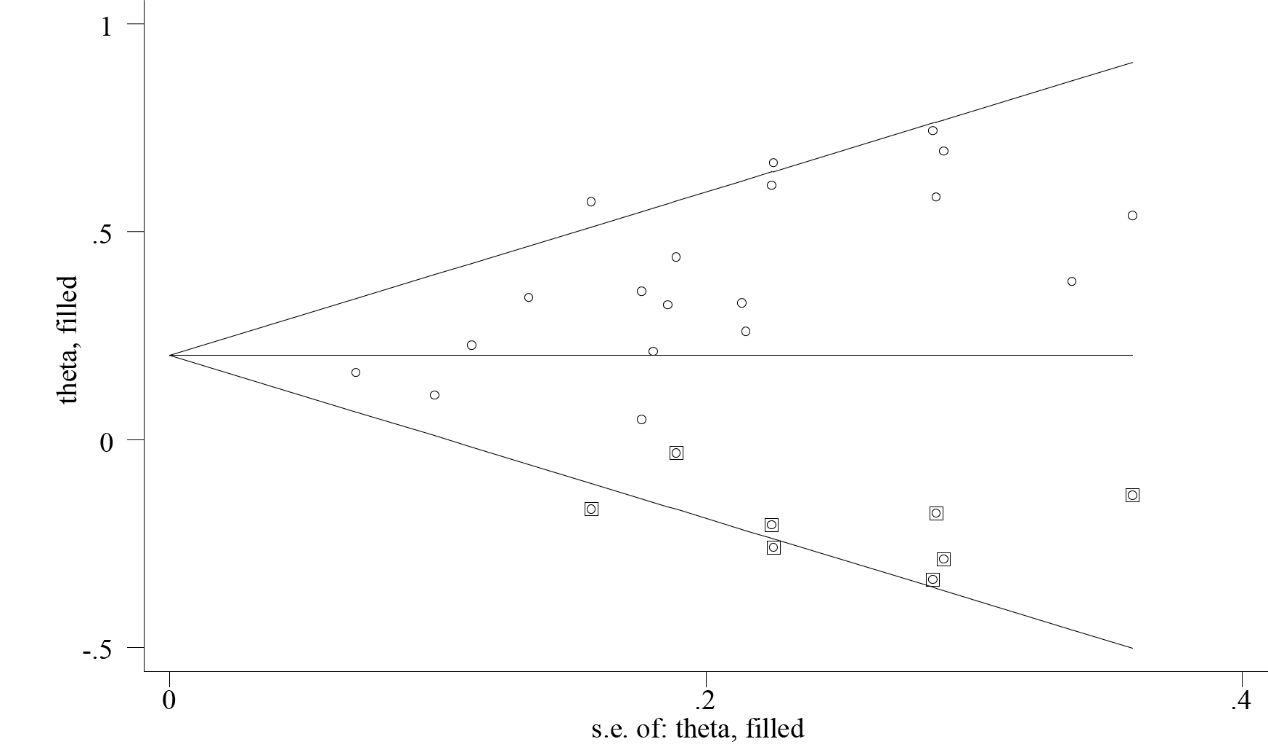


Figure S14 Cut-and-patch method illustration for 3-year survival rate


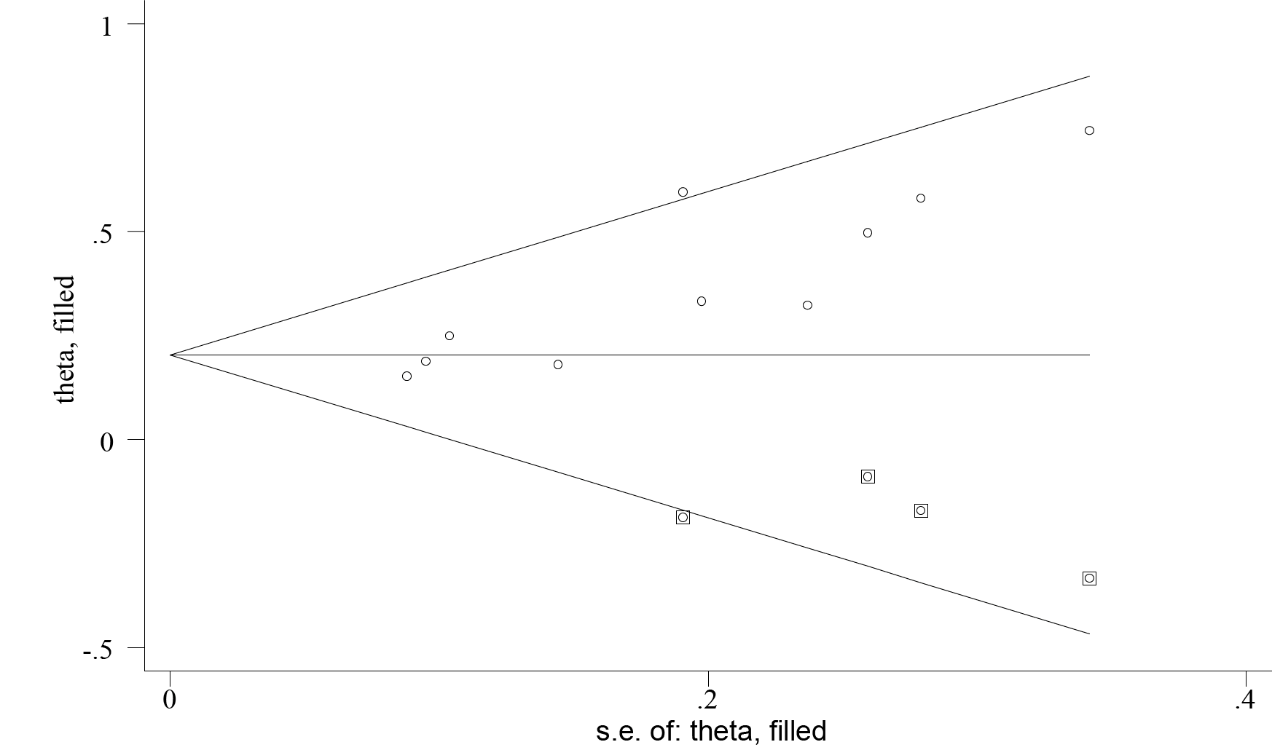


Figure S14 Cut-and-patch method illustration for chemotherapy disruption rate


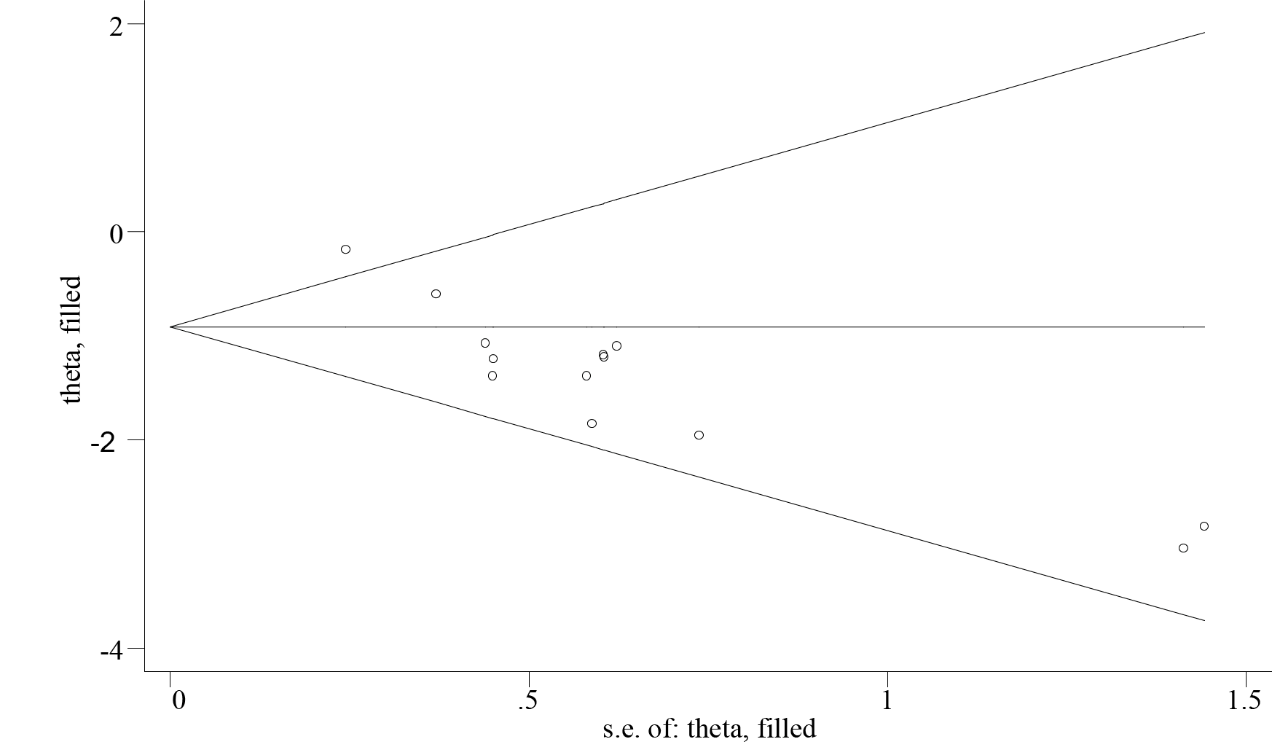

Supplement: Supplementary file 1 [file DataSheet_1.docx]
